# Biased gene expression in early honeybee larval development

**DOI:** 10.1186/1471-2164-14-903

**Published:** 2013-12-19

**Authors:** Rosannah C Cameron, Elizabeth J Duncan, Peter K Dearden

**Affiliations:** 1 Laboratory for Evolution and Development, Gravida, the National Centre for Growth and Development and Genetics Otago, Department of Biochemistry, University of Otago, Dunedin, Aotearoa-New Zealand

**Keywords:** Caste development, Larval development, Gene expression, *Apis mellifera*

## Abstract

**Background:**

Female larvae of the honeybee (*Apis mellifera*) develop into either queens or workers depending on nutrition. This nutritional stimulus triggers different developmental trajectories, resulting in adults that differ from each other in physiology, behaviour and life span.

**Results:**

To understand how these trajectories are established we have generated a comprehensive atlas of gene expression throughout larval development. We found substantial differences in gene expression between worker and queen-destined larvae at 6 hours after hatching. Some of these early changes in gene expression are maintained throughout larval development, indicating that caste-specific developmental trajectories are established much earlier than previously thought. Within our gene expression data we identified processes that potentially underlie caste differentiation. Queen-destined larvae have higher expression of genes involved in transcription, translation and protein folding early in development with a later switch to genes involved in energy generation. Using RNA interference, we were able to demonstrate that one of these genes, *hexamerin 70b*, has a role in caste differentiation. Both queen and worker developmental trajectories are associated with the expression of genes that have alternative splice variants, although only a single variant of a gene tends to be differentially expressed in a given caste.

**Conclusions:**

Our data, based on the biases in gene expression early in development together with published data, supports the idea that caste development in the honeybee consists of two phases; an initial biased phase of development, where larvae can still switch to the other caste by differential feeding, followed by commitment to a particular developmental trajectory.

## Background

Caste development in honeybees (*Apis mellifera)* is a remarkable example of, and model for, developmental plasticity. In this species, two adult female phenotypes are produced by one genome in response to diet [1]. Larvae destined to become queens are fed royal jelly (RJ) which is rich in carbohydrates and lipids [2]. RJ also contains major royal jelly proteins, one of which, royalactin, is vital for queen development [3]. As a result of a higher food intake [4] and a more nutritious food, queen bees are larger, longer-lived, have fully developed ovaries and differ in their behaviour and morphology, when compared to worker bees. Transferring larvae from worker cells to queen cells (thus manipulating their exposure to RJ) during early larval development causes bees to develop some queen-like phenotypes. Based on these experiments it has been proposed that differentiation between the two castes begins on the first day of larval development and is progressive [5]. Manipulating larval diet in this way can induce queen-like properties in worker bees up to 60 hours of larval development [6] demonstrating, that at least during early larval development, there is plasticity in the developmental pathways that give rise to queen and workers. The molecular mechanisms by which RJ triggers queen development are only beginning to be understood, but RJ intake causes differences in juvenile hormone (JH) titre between worker and queen larvae [7]. These JH levels regulate gene expression [8]. It seems likely, therefore, that a complex set of molecular mechanisms link RJ exposure to changes in JH titre. Such mechanisms should be reflected in differences in gene expression between developing larvae destined for different castes.

Differences in gene expression underlying queen and worker developmental trajectories have been investigated in a number of previous studies [8-16]. These studies used a variety of techniques to profile gene expression, but have focused on mid-to-late larval development as few differences in gene expression have been identified before this stage [15]. Mid to late larval development includes the period (larval stage 3 and 4), when JH titres differ between castes [7], and so if is difficult to determine if these gene expression changes are due to RJ exposure, or the action of JH.

While a number of mechanisms that regulate gene expression during caste development have been proposed, DNA methylation is thought to be critical, as it can link environmental exposure to changes in gene expression [17]. RNA interference targeting the *de-novo DNA methyltransferase 3* (*Dnmt3*) biases development towards the queen trajectory [18]. Honeybees, similar to other invertebrates, have relatively low overall levels of CpG methylation restricted to gene bodies [19,20]. Methylation within the gene body appears to influence alternative splicing in the honeybee [19,21-23] and the ant [24]. It is possible that the role of DNA methylation in caste development may be via influencing alternative splicing of transcripts. Evidence from mammalian models indicates that gene body methylation may have other molecular functions, such as promoting RNA polymerase pausing [21], modulating other epigenetic marks [25,26], or affecting altering promoter usage [26]. It has been proposed that plasticity, such as caste development in eusocial insects and neuronal plasticity in invertebrates, is associated with dynamic changes in DNA methylation [27-29] and, by inference, alternative splicing. DNA methylation is likely to be one of many molecular mechanisms involved in caste development, but these mechanisms as a whole will lead to changes in gene expression, detectable using microarray techniques, or alternative splice isoforms detectable using transcriptomics.

We have examined gene expression throughout larval development, both before and after the wave of JH, in queen and worker castes, carrying out the widest survey of gene expression during caste development in this remarkable animal. We used this to build a more complete understanding of how a honeybee larva responds to RJ, and how this biases them into an alternative developmental trajectory, culminating in a queen bee.

## Results and discussion

### Experiment design

There are five stages of larval development (L1-L5) in the honeybee. Queen and worker samples were taken at the following time points (the corresponding larval stages are shown in brackets): 6 hours (L1), 12 hours (L1), 36 hours (L2), 60 hours (L3), 84 hours (L4), 108 hours (L5) and 132 hours (L5). At each time point four replicate queen and worker samples were collected. Each replicate sample collected at 6 hours contained 20 larvae and samples collected at all other time points contained 5 larvae.

Queen development was induced by grafting larvae (transferring newly emerged larvae with a small paintbrush) into artificial queen cells before returning them to the hive. Grafting, which is a standard apicultural technique, is carried out as soon as practical after the larvae hatch (within ~1 hour). Larvae in these queen cells are fed RJ and this triggers queen development. When collecting 6-hour larvae it was noted that RJ was present in these cells. Worker larvae were not grafted. Gene expression data was generated from these samples and using custom two colour long-oligonucleotide microarrays (13,440 double spotted oligos representing 13,135 sequences). At 60 hours, two additional biological replicates, each consisting of either 20 queen or worker larvae, were taken and transcriptome data was generated using high throughput sequencing (HTS). The 60 hours time point was selected for HTS as this time point has been highlighted as the earliest difference in gene expression seen a previous study [15]. HTS sequencing also gave us the opportunity to assess gene expression in greater depth, as well as analyse the effect of caste development on alternative splicing.

#### Differential gene expression during caste development

Microarray analysis indicates that queens and workers have relatively equal numbers of differentially expressed genes (DEGs) throughout larval development (Table 1). HTS of RNA, however, found that 83 % of DEGs had higher expression in queens, similar to previous results [13]. Many studies have reported finding more differentially expressed genes using HTS than using microarrays [30-33]. This is at least partly because HTS analysis is not hindered by the acquisition bias that can affect microarray analysis. Microarrays are sensitive to saturation effects and are also not able to detect transcripts that are expressed at a relatively low level due to high amounts of background fluorescence obscuring low hybridization signal to the probes. We believe the latter phenomena accounts for the relatively high number of DEGs detected in queens by HTS compared to microarray, as these genes had a median RPKM of 51.25 compared with a median RPKM of 150.65 for genes that are more highly expressed in worker larvae. We believe the relatively low expression of these genes precluded them from being detected as differentially expressed by microarray analysis. We have carried out extensive validation of these data sets using RT-qPCR (Additional file 1: Figure S1) which indicates that both the microarray and HTS experiments are producing high-quality, biologically relevant information.

**Table 1:** Number of differentially expressed genes between queen and worker larvae

	**6 hr**	**12 hr**	**36 hr**	**60 hr**	**84 hr**	**108 hr**	**132 hr**	**60 hr HTS**
Queens	442	76	154	327	330	250	115	2311
Workers	556	99	156	304	362	197	160	457
Total	998	175	310	631	692	447	275	2768

Genes we identified as being differentially expressed during caste development were assigned *Drosophila* orthologs using BlastX [34]. Approximately 90 % of the genes with higher expression in queen larvae have orthologs in *Drosophila* as compared with 77 % of the genes with higher expression in workers, a statistically significant difference (*P* < 2.2 × 10^-16^, Fisher’s exact test) (Additional file 1: Table S2). Barchuk *et al.* (2007) also found a tendency for worker larvae to have higher expression of genes that lack orthologs in *Drosophila*[8]. These data imply that genes more highly expressed by queen larvae have been conserved across ~350 million years of evolution. In contrast, genes more highly expressed in worker larvae are not as well conserved in *Drosophila* and may be rapidly evolving.

Differences in gene expression between queen and worker larvae are observed as early as 6 hours (L1) post-grafting (Table 1). Indeed, the largest numbers of DEGs identified by microarray analysis are detected at this time point (Table 1). While it is possible that the large number of differentially expressed genes at this time point are due to perturbation of the larvae during grafting, the appearance of genes that are differentially expressed at 6 hours and that retain higher expression in one caste during larval development implies that at least some of this differential gene expression represents an early response to RJ. We also do not observe gene ontology categories associated with stress response or physiological perturbation in the genes that are differentially expressed at 6 hours, indicating that differences we observe in expression at this early time-point are likely an early response to RJ.

In queen larvae, several genes with increased expression early in larval development are maintained at high levels throughout larval development. These genes are *mitochondrial cytochrome C*, *phosphoenolpyruvate carboxykinase* (PEPCK), *phytanoyl-CoA dioxygenase domain-containing protein 1 homolog* and *glycine N-methyltransferase-like* (GNMT). Worker larvae, in contrast, have higher levels of the apoptosis regulator *Bcl-2* throughout development. These genes have functions that are difficult to interpret in terms of caste development, but are consistent markers of either worker or queen development.

Given that queen development can be triggered in larvae by grafting until larval day three, well after the 6 hour time point by which we see substantial differences in gene expression, these early differences in gene expression must not commit the larvae to either a queen or worker fate. We therefore propose these early changes in gene expression, in response to RJ, may bias development toward one caste or the other, but that later gene expression can reverse that bias. These early differences in gene expression may be attributable to the larval diets which differ, with the queen diet initially containing more sugar [35] and less protein [36] than worker jelly [reviewed in 4] though it is not clear if these differences occur at or before 6 hours after grafting. These dietary differences have also been linked to morphological differences between the castes that are apparent as early as 2 days into larval development [5]. The idea of an early, biased, phase of caste development is consistent with early larval grafting experiments which showed that worker larvae shifted into queen cells at ~12 hours of larval development result in bees that are queen-like, but retain some worker characteristics [5]. A two-stage process of caste development has been previously proposed based on grafting, nutrition and physiological data [5], but our data provides the first molecular evidence to support an early biased phase of gene expression and development followed by a commitment phase.

#### Differential expression of hormone biosynthesis genes

One key event during larval development is the wave of JH synthesis in queen larvae, during L3 and L4 stages [7]. Juvenile hormone (JH) is implicated in caste development in honeybees [37]. Our data indicates that several genes involved in JH and ecdysteroid biosynthesis are differentially expressed during larval development between castes (Additional file 1: Table S3) at, or around this JH wave. Queen destined larvae, have higher expression of eight CYP genes, many with roles in hormone biosynthesis. *CYP315A1,* acts in ecdysteroid biosynthesis [38], and has higher expression at six hours of larval development. *CYP15A1,* which has higher expression in queens throughout larval development but peaking at 84 hours, acts as a JH epoxidase, catalysing the final step of JH biosynthesis [39].

Queens also have higher expression of JH methyltransferase, involved in the final steps of JH synthesis, with differential expression peaking at 84 hours after grafting (Additional file 1: Table S3). In contrast, during mid larval development, workers have higher expression of JH esterase, which degrades JH in honeybees [40]. Seven genes induced in *Drosophila* by JH titre [41] are dynamically regulated, with three having higher expression in workers early in development and five having higher expression in queens during mid to late development, temporally correlating with the peak of JH titre during development [7].

**Figure 1:**
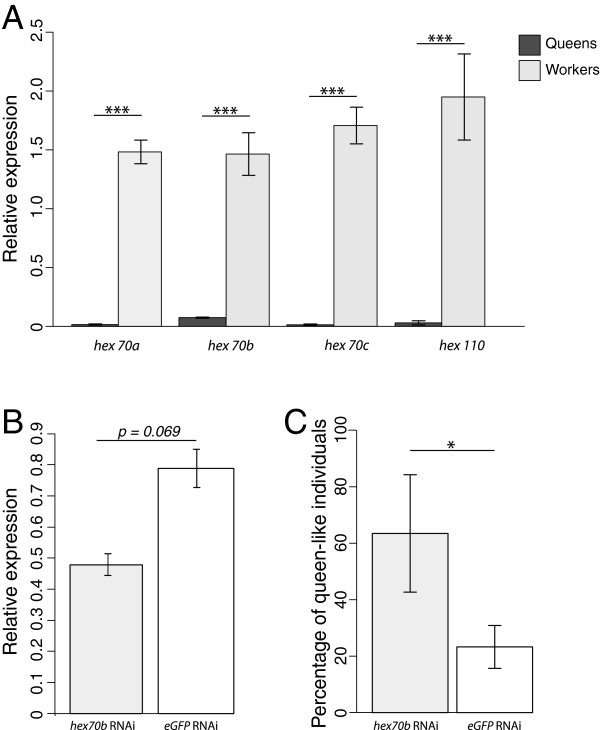
**Model depicting the major findings from this study.** In this study we have identified substantial differences in gene expression that can be detected as early as 6 hours after exposure to RJ. As indicated by the arrows, a small number of these genes maintain higher expression throughout larval development in either the queen caste (red arrow, *mitochondrial cytochrome C*, *phosphoenolpyruvate carboxykinase* (PEPCK), *phytanoyl-CoA dioxygenase domain-containing protein 1 homolog* and *glycine N-methyltransferase-like* (GNMT)) or worker caste (blue arrow, *Bcl-2*). The differences in gene expression occur earlier in larval development than previously reported [8,15] and before the point at which we know larvae become committed to a particular caste [6]. These early, and sustained, differences in gene expression have led us to propose that this phase of early development represents a biased developmental trajectory. During this phase of early larval development gene expression is altered in response to RJ, biasing towards queen development (red line), or worker development (blue line) but not irreversibly so. Following this period, corresponding with peak JH levels in queens, we observe unique sets of genes induced in queen (red arrow, *juvenile hormone-inducible (Jhl-26)*) and worker castes (blue arrow, *translocator protein, shep-like, lethal(2) essential for life and LOC10058039*) that remain stably more highly expressed throughout the remainder of larval development. We propose that these changes in gene expression at 84 hours of larval development are associated with the induction of a committed developmental trajectory.

The trends in hormone-related gene expression in queen larvae, peaking as they do during mid-late larval development, are consistent with these genes either being involved in the synthesis of JH associated with the wave between the third and fourth larval instars, or with responding to the JH wave. At this point we also observe the appearance of differential expression of a number of genes in both queen and worker larvae that remain stably differentially expressed throughout the remainder of larval development (Figure 1). This change to a committed developmental trajectory, however, does not alter or reprogram the expression of genes we find differentially expressed at early stages. Our data is consistent with an initial bias towards one caste or the other induced quite early (by 6 hours) by RJ consumption, followed by a consolidation of that fate and final specification through circulating hormones.

#### Differential expression of Cytochrome P450 genes

**Figure 2:**
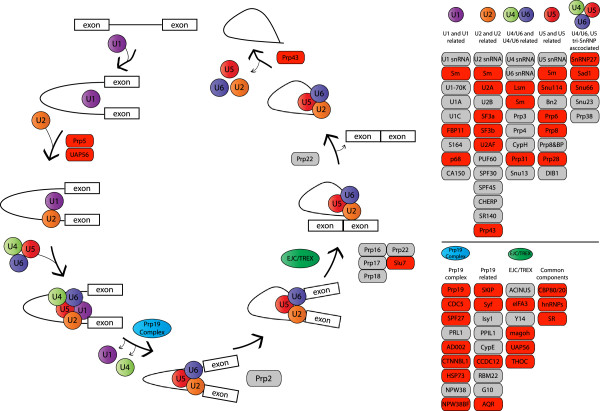
**The CYP gene family is extensively differentially regulated during caste development.** CYP genes are listed below the caste and time-point where they are differentially expressed. For clarity only CYP genes that are differentially expressed are shown. Genes are colour coded according to the clan they are assigned to based on based on sequence similarity [38]. The total number of CYP genes identified in the honeybee genome for each of the four clans [38] is indicated beside the colour key.

*Cytochrome P450* (*CYP*) genes (Figure 2) which act in hormone and pheromone biosynthesis [42] and the detoxification of foreign compounds [38] show differential regulation between castes. Insect *CYP* genes are divided into four major clans based on sequence similarity [43]. Worker larvae have higher expression of 18 *CYP* genes at time points throughout larval development, the majority belonging to clan three, in particular sub-families six and nine. There is evidence for rapid expansion of these two sub-families in the honeybee through recent gene duplication events [44]. Genes in CYP clan three are loosely classified as environmental response genes [38].

The differential expression of CYP clan three genes is most marked at 60 hours of larval development, where worker larvae have higher expression of 13 genes of this clan. This period coincides with a change in the worker larval diet which results in the introduction of pollen grains, whereas RJ only contains trace levels of pollen [4]. The introduction of pollen exposes worker larvae to a number of foreign compounds. One example of this is the addition of quercetin, a flavinoid compound found in honey and pollen [45]. Four CYP genes are known to metabolise quercetin in the honeybee (CYP6AS1, CYP6AS3, CYP6AS4 and CYP6AS10) [45] and all four have higher expression in workers during mid-larval development. Some CYP genes are involved in pheromone production [44] and possibly caste specific synthesis of mandibular acids [46]. CYP genes have also been linked to termite caste development [47,48].

### Functional classes of genes differentially regulated between queen and worker larvae

Two other families of genes were differentially expressed during caste development (Additional file 1: Table S3). Queens have higher expression of five genes involved in the response to reactive oxygen species (ROS), especially during mid larval development. These genes include catalase, several thioredoxins and a peroxiredoxin. The generation of ROS by the mitochondria has been proposed to be a key mechanism in the aging process [49]. This may reflect an increased respiration rate previously observed in queen larvae [50]. The differential regulation of these genes may influence expression of *vitellogenin*, important in protecting honeybees from oxidative stress [51]. In contrast workers have higher expression of three glutathione-s-transferase genes. Glutathione-s-transferases are important for detoxification of both endogenous (ROS) and exogenous (xenobiotic) compounds [52].

Programmed cell death has a role in reducing ovariole numbers in larval worker ovaries during fourth and fifth larval instars [53,54]. Consistent with this we see higher expression of eleven genes involved in programmed cell death in worker larvae. These belong to two major programmed cell death pathways - apoptosis and autophagic cell death.

### Hexamerin70b and caste development

**Figure 3:**
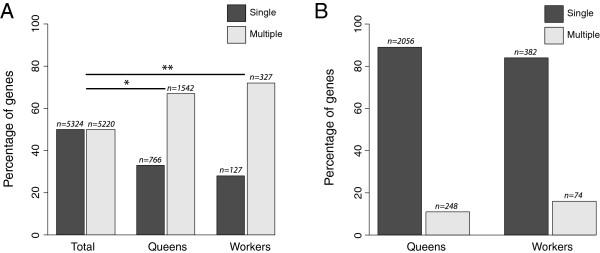
***Hexamerin 70b *expression biases caste determination towards worker fate. A.** Relative expression of the four hexamerin genes in 60 hour queen and worker larvae as determined by RT-qPCR. Error bars represent the standard error of the mean. Statistical significance was assessed using a one-way ANOVA, *** indicates P < 0.001. **B.** Relative expression of *hex70b* was determined 12 – 18 hours following injection of either *hex70b* or *eGFP* (control) dsRNA using RT-qPCR. Error bars represent the standard error of the mean. Statistical significance was assessed using a one tailed t-test. **C.** The phenotype of emerging bees was assessed using a suite of morphological characteristics (refer to Additional file 1 for more detail). Graph shows the percentage of individuals that developed as queen-like after RNAi against *hex 70b* or *eGFP* (control). Statistical significance was assessed using a t-test, * indicates P < 0.05.

Hexamerin genes were differentially regulated between castes at several larval time-points. RT-qPCR [55], which is generally considered to be the gold-standard method to measure gene expression [56], was used to confirm that, at 60 hours of larval development, workers have between 20 and 125 fold higher expression of the four hexamerin genes when compared to queens (Figure 3A). Hexamerins are a family of insect amino acid storage proteins that have evolved from the copper-containing hemocyanins of ancestral aquatic insects and crustaceans [57]. A role for the hexamerins has been suggested in caste development in termites, where two hexamerin genes have been shown to facilitate the JH dependent worker to soldier differentiation [58]. Several previous studies have found differences in expression of hexamerin genes between queens and workers implying a possible role for these proteins in caste development [11,15,59].

*Hexamerin 70b* (*hex 70b*) is expressed in the fat body and gonads during larval development and its expression, which is regulated by juvenile hormone [59,60], peaks prior to the expression of other hexamerins during larval development [59]. Thus far no biological function has been attributed to hex 70b; it is found in the cytoplasm, nucleus, and in punctate dots in the cytoplasm of cells within the fat body [61], implying that this protein has multiple biological roles, as reported for hex 70a [62].

### Hexamerin70b knock-down

Of the four hexamerins assayed by RT-qPCR (Figure 3A) *hex 70b* showed the smallest fold change between queen and worker larvae (25-fold, Figure 3A). This comparatively small fold-change provides us with the best opportunity, amongst the hexamerins, to knock-down expression of this gene to queen larvae-like levels, even if RNAi treatment is inefficient and knock-down is weak. Given the technical challenge of RNAi in honeybee larvae, and the high expression levels of all the hexamerin genes, we targeted *hex 70b* because moderate knockdown of this gene was more likely to produce queen-like expression levels, and give a clear, measurable, phenotype. RNAi knockdown of *hex 70b* resulted in significant changes (*P* = 0.04) in the proportion of queen-like individuals produced when compared to control injected larvae (Figure 3C). Emerging individuals were classified as queen-like based on the morphology of several key structures (see Additional file 1). While *hex 70b* RNAi produced a statistically significant change in phenotype, we did not see a significant difference in the expression of *hex 70b* between target and control larvae as determined by RT-qPCR (Figure 3B), although we did observe a trend towards lower *hex 70b* expression in the hex 70b RNAi injected group relative to controls. The lack of statistical significance likely reflects the small sample sizes used for RT-qPCR, and large variation in expression of this gene in injected individuals. Samples were taken 12 – 18 hours post-injection for this analysis and it is possible that sampling too early, or too late, may explain why the difference in expression between the target and control groups was not significant.

Knockdown of *hex 70b* biases individuals towards a queen fate (Figure 3C), consistent with the high expression of these genes in worker larvae (Figure 3A). We propose that *hex 70b* has a key role in caste development at a time when we propose a commitment to worker or queen fate is set (Figure 1). This implies that early biases in gene expression between worker and queen larvae can be overcome to change the developmental trajectory of a larva. The possibility that hexamerins are acting to regulate JH activity [63] raises the possibility that we are disrupting the final commitment phase of caste development by reducing *hex 70b* expression, rather than changing the bias in commitment.

#### Gene ontology and pathway analysis

DEGs involved in caste development were categorized by proposed gene function by examining enriched Gene Ontology (GO) terms, (Additional file 1: Table S4) and pathways (Additional file 1: Table S5) taking advantage of well annotated *Drosophila* orthologs of these genes. This analysis further reinforces the idea of unique caste-specific developmental trajectories in female honeybees; queen and worker larvae show different trends in terms of the pathways and processes that are more highly expressed during development. Differential activation of these pathways and processes are likely to reflect the different requirements of the adult phenotypes.

During early development queen larvae have higher expression of genes in pathways involved in cellular maintenance and growth. Genes involved in DNA replication and amino acid metabolism pathways are enriched at 6 hours, and GO terms include nucleotide binding, protein folding and ATPase activity. During mid larval development queen larvae have higher expression of genes involved in energy generation pathways, including the tricarboxylic acid cycle (TCA) and oxidative phosphorylation. This switch to energy generating processes at 84 and 108 hours may reflect in the increased growth rate seen in queen larvae. Worker larvae are heavier than queen larvae up until 84 hours, when queens begin to gain weight faster than workers [64]. In late larval development, queens have higher expression of genes with GO terms associated with proteolysis.

Workers have higher expression of genes involved in amino acid, xenobiotic and general metabolism from early through to mid development. Workers also have higher expression of genes involved in muscle development early in larval development. The higher expression in workers of genes involved in muscle development was also noted by Barchuk *et al.* (2007) [8]. Barchuk *et al.* (2007) suggest that this may represent worker larvae preparing for their adult life as foragers. As the thoracic muscles disappear entirely during metamorphosis [65], this investment must be able to be carried through to adult muscle development to be beneficial to flight muscle strength.

Similar to queens, workers have higher expression of genes involved in proteolysis during late larval development; however, in workers these genes have higher expression at 108 hours in contrast to 132 hours in queens. This temporal shift in gene expression was not a common phenomenon in our data sets (data not shown). The difference in expression of these proteolysis genes may reflect preparation for pupation in queen larvae. In workers, at 108 hours, the expression of these genes is unlikely to be related to pupation but instead may have a role in mediating PCD in the worker ovary [66]. Consistent with this hypothesis we see higher expression of genes involved in apoptotic (cathepsins) and autophagic cell death (*thread* and *Ice*) in workers during mid to late larval development (Additional file 1: Table S3).

#### Alternative splicing of mRNA transcripts and caste development

**Figure 4:**
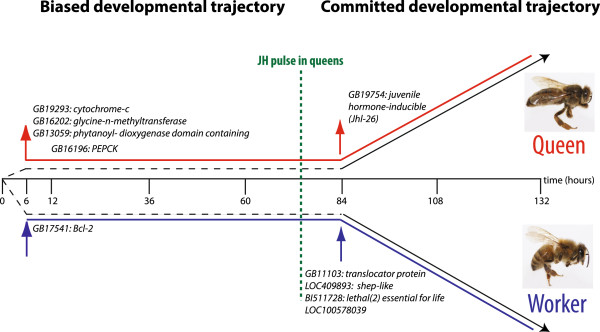
**Annotation of genes involved in the spliceosome showing genes that are more highly expressed in queen larvae.** The left hand side of the figure depicts the assembly of the spliceosome on a primary mRNA. Genes encoding proteins involved in the spliceosome are depicted as oblong boxes, with those more highly expressed in queen larvae shown in red and the remaining genes in grey (no genes were more highly expressed in worker larvae). The five small nuclear ribonucleoproteins that make up the spliceosome are depicted as circles (U1-U5). The spliceosome associated complexes Prp19 and EJC/TREX and are also shown.

One set of genes with consistently higher expression in queens were those associated with the spliceosome (Figure 4). This is particularly interesting as spliceosome encoding genes are differentially methylated between castes [22], and a link has been proposed between gene-body methylation and control of alternative splicing in the honeybee [19,21-23] and the ant [24].

DNA methylation is believed to be crucial to caste development [18,67] as RNA interference targeting the *de-novo DNA methyltransferase 3* (*Dnmt3*) biases development towards the queen trajectory [18]. Honeybees, similar to other invertebrates, have relatively low overall levels of CpG methylation restricted to gene bodies [19,20]. Over evolutionary time methylated cytosines are more often deaminated to thymines than non-methylated cytosines [68], leaving a signature of low CpG content in methylated gene bodies (which are preferentially methylated in invertebrates [20]). The CpG content of a gene (as measured by the observed versus expected ratio of CpG dinucleotides in a coding sequence or CpG_[o/e]_) can be used to infer the ancestral germ-line methylation state of a gene [69]. While CpG_[o/e]_ ratio does not indicate the current methylation state of a gene, several studies have found good correlation between low CpG_[o/e]_ values and high DNA methylation in the honeybee [19,70,71]. Analysis of the CpG_[o/e]_ ratios for the DEGs implies a dynamic pattern of switching between historically methylated and historically un-methylated transcripts as development proceeds (Additional file 1: Figure S6H). If current DNA methylation is related to CpG_[o/e]_ measurements, as suggested by previous studies, then our data implies a complex and changing relationship between methylation and gene expression during caste development.

DNA methylation of introns and exons has been associated with alternative splicing of mRNA transcripts in diverse species [22,23,72,73]. Differences in DNA methylation have been detected during larval development [74] and knockdown of *Dnmt3* has been shown to cause aberrant splicing in honeybee larvae [75]. Given the proposed link between alternative splicing and methylation in the honeybee [19,21-23], and our evidence implying queens have higher expression of spliceosome related genes (Figure 4) we used our HTS dataset to investigate alternative splicing.

**Figure 5:**
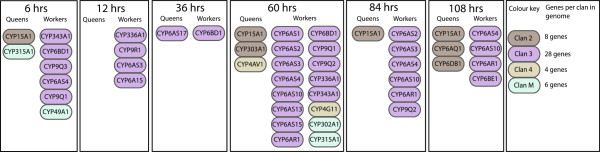
**Association of differentially expressed genes with alternative splicing. (A)** Percentage of genes encoding single (dark bars) and multiple transcripts (light bars) as determined by HTS at 60 hours of larval development. Statistical significance was assessed using a Chi-squared test, * P < 0.05, ** P < 0.01. The number of genes in each category is indicated on the graph. **(B)**. Percentage of DEGs where a single transcript (dark bars) or multiple transcripts (light bars) are differentially regulated. The number of genes in each category is indicated on the graph.

Discovery of alternative transcripts in our datasets indicates that ~50 % of genes expressed in larvae have alternative transcripts. For DEGs both queens and workers have higher expression of more genes than expected that encode multiple splice variants in larvae (*P* = 0.02 and 0.002) (Figure 5A). However, both queens and workers tend to show higher expression of a single variant only rather than multiple variants of the same gene (Figure 5B). Thus in the majority of cases a single transcript is differentially regulated, while the other transcript(s) for these genes remain constitutively expressed. There are, of course, exceptions to this; in queens 11 % of DEGs and 16 % in workers have multiple transcripts that are differentially regulated. In all cases, bar three, these genes are regulated co-ordinately. For three DEGs, splice variants are regulated independently, *i.e.* one transcript of the gene is more highly expressed in queens, another in workers. These genes encode a DNAJ-type chaperone (*LOC408966*), a RNA exonuclease (*LOC413245*) and a TLD-domain containing protein (*LOC100576519*). While queens and workers both have higher expression of genes that have the capacity to encode multiple splice variants, usually the transcription of only a single variant is affected.

Our analysis indicates that while alternative splicing is abundant in both queen-destined and worker-destined larvae, we find little evidence for alternative splicing contributing to the queen and worker developmental trajectories. That we find specific transcripts of genes that are differentially expressed, against a background of invariant expression of other splice variants, implies that alternative splicing may play a role in caste development, but that this role is neither a simple one nor limited to a specific caste. The increase in spliceosome gene expression may reflect an increase in general transcription, with queens expressing more genes than workers, as shown in our HTS data.

Alternative splicing has recently been linked to DNA methylation in the honeybee [19,21-23,75] and the ant [24]. Until recently most of the evidence for a link between alternative splicing and methylation in the honeybee implies that methylation promotes exon skipping [19,22], although evidence exists for promotion of exon inclusion [23,75]. In general, however, alternative transcripts occur more often in methylated genes as compared with unmethylated genes [23]. We know that DNA methylation is integral for worker development [18] and our data, that alternative splicing does not correlate with a particular developmental trajectory, hints at a role for DNA methylation in caste development outside the regulation of alternative splicing.

## Conclusions

Caste development in honeybees is a complex process involving coordinated changes in gene expression triggered by larval diet. Our study indicates that these changes occur earlier than previously thought, implying an early biasing of gene expression in each developing caste, followed by a commitment phase which may be triggered by circulating JH (Figure 1).

Both castes are likely to have changed from the non-social ancestor of honeybees, however we propose that the gene expression profile accompanying the queen developmental trajectory, based on the evolutionary history of the genes expressed, is more similar to that seen in the ancestral non-social bee. The gene expression profile accompanying the worker developmental trajectory represents genes, and gene networks, that are more diverged from this ancestor implying that novel genes and pathways have been co-opted in worker development into regulating this complex polyphenism.

## Methods

### Sample collection

*A. mellifera* larvae were collected from single-drone inseminated queen bees from a closed population of bees (Betta Bees Research Ltd). A subset of newly hatched larvae were grafted (moved with a paintbrush) into queen rearing cells and placed in a queen-less hive. The remainder of larvae were placed in a queen-right hive and raised as workers. Larval samples were collected at 6 hours, 12 hours, 36 hours, 60 hours, 84 hours, 108 hours and 132 hours of larval development. At each time-point four replicate queen and worker samples were collected. Each of the four samples collected at 6 hours contained 20 larvae. At later time-points each sample contained 5 larvae. Samples were snap frozen in liquid nitrogen and stored at −80 °C.

### RNA extraction

Total RNA was extracted from larval samples using TRIZOL® (Invitrogen), and purified using RNeasy columns (Qiagen). RNA integrity was confirmed by gel electrophoresis.

### Microarray detection of differential gene expression

The honeybee microarrays used for this experiment were custom spotted long oligonucleotide microarrays available from the University of Illinois (NCBI GEO Platform GPL15631 (13,440 double spotted oligos representing 13,135 sequences)). At each of the seven time-points four replicate microarrays were performed with dye swaps for two of the four microarrays. 10 μg of total RNA from each sample was amplified and labelled using the Ambion Amino Allyl MessageAmp™ II aRNA Amplification kit. The aRNA was labelled with Alexa Fluor dyes 647 or 555, fragmented (Ambion fragmentation buffer) and hybridised to microarray slides (Ambion slide hybe buffer). Post hybridisation, microarray slides were washed in four wash solutions with increasing stringency before being scanned. Microarrays were scanned with a GenePix 4000B Microarray Scanner (Molecular devices) and images processed, to determine intensity values for each gene in each condition, with the GenePix Pro 4 software (Molecular devices). GenePix results files were analysed with GeneSpring GX 10 (Agilent) using the advanced analysis pathway for analysing generic two-colour microarray data. Lowess normalisation was performed on the data with no baseline transformation. Probes were filtered by flags that were assigned in GenePix Pro 4.0. Any spots flagged as marginal or absent were removed from the analysis. Probes were then filtered by expression with a cut-off of 1000. The statistical significance of differential gene expression was assessed with an unpaired t-test and a P-value of less than 0.05 was considered significant.

### High throughput sequencing (HTS) detection of differential gene expression

Two biologically independent replicate samples of 10 μg of queen and worker RNA from 60-hour larvae were subjected to RNA-seq analysis on a Illumina HiSeq 2000 (service provided by Beijing Genomics Institute). Each of the four samples yielded between 6.97 and 7.37 million 50 bp reads. The reads were filtered and high quality sequence reads were assessed for differentially expressed genes using CLC Genomics Workbench platform software (CLC bio). The RNA-seq tool and *Ab Initio* transcript discovery plug in were used to identify all differentially expressed and alternatively spliced transcripts. Sequence reads were mapped back to the honeybee genome (Amel_4.5, NCBI) with 85 % of reads mapping to 9578 genes (of the 11156 annotated genes in the honeybee genome). The RPKM (Reads Per Kilobase of exon model per Million mapped reads) was calculated for each transcript. RPKMs were normalized between biological replicates using scaling normalization [76]. Differentially expressed genes (DEGs) were identified using the Baggerly test, which is similar to a two sample t-test but the test statistic is weighted according to the number of reads in each sample [77], as implemented by CLC Genomics Workbench. Genes that had a false discovery rate corrected P values of less than 0.05 were considered to be differentially expressed. The data discussed in this publication have been deposited in NCBI’s Gene Expression Omnibus [78] and are accessible through GEO Series accession number GSE52291 (http://www.ncbi.nlm.nih.gov/geo/query/acc.cgi?acc=GSE52291). *Drosophila* orthologs of the DEGs were obtained using blastx within Blast2GO using default parameters (E value cut-off of 1 x 10^-3^) [79] (Additional file 2). To determine if the microarray and HTS data was of a standard useful for large scale analyses, such as Pathway or Gene Ontology (GO) analysis, several quality control assessments, including extensive quantitative RT-PCR, were performed (Additional file 1: Figure S1).

### Pathway and GO analysis

The enrichment of potential functions of *Drosophila* orthologs of candidate genes identified in the microarray and HTS was identified using the Database for Annotation, Visualisation and Integrated Discovery (DAVID). The background list used for the analysis of the DEGs from the microarrays consisted of the *Drosophila* orthologs of all genes present on the microarray. The HTS background list consisted of the *Drosophila* orthologs of all the genes expressed in larvae at 60 hours. For pathway analysis a P-value cut off of 0.05 was used. The GO cluster analysis was used and any clusters with enrichment scores below 1.0 were discarded.

### Quantitative RT-qPCR

One μg of RNA from independent biological replicate samples of the 12-hour, 60-hour, 84-hour and 108-hour time-points was used to make cDNA using Superscript® VILO™ according to manufacturer’s instructions. Superscript® VILO™ uses random primers to prime first stranded cDNA synthesis, which is recommended for RT-qPCR as it allows for more representative sampling of the mRNA [80]. Oligonucleotide primers were designed using Primer3 [81] and Amplify [82]. Quantitative RT-PCR, normalization and data analysis was performed as previously described [55].

### RNAi

dsRNA was synthesised for *hex 70b* and *eGFP* (control) using the Ambion MEGA®script RNA kit. The dsRNA probe spanned exons six and seven of the *hex70b* transcript. RNAi and larval rearing methods were based on those described in Beye *et al.* 2002 and Kucharski *et al.* 2008 [18,83]. To ensure that results were consistent the RNAi experiment was repeated on three independent occasions (total number of individuals = 43 for *Hex70b* RNAi and 48 for *eGFP* injected controls). For more information see Additional file 1 section.

### Availability of supporting data

The data discussed in this publication have been deposited in NCBI’s Gene Expression Omnibus and are accessible through GEO Series accession number GSE52291 (http://www.ncbi.nlm.nih.gov/geo/query/acc.cgi?acc=GSE52291).

## Abbreviations

DEGs: Differentially expressed genes; GO: Gene ontology; HTS: High throughput sequencing; JH: Juvenile hormone; RJ: Royal jelly; ROS: Reactive oxygen species.

## Competing interests

The authors declare that they have no competing interests.

## Authors’ contributions

RCC performed the majority of the experiments and contributed to writing the manuscript. EJD assisted with larval RNAi, analysis of experimental data and writing of the manuscript. PKD planned and carried out some of the experiments and contributed to drafting the manuscript. All authors read and approved the final manuscript.

## Supplementary Material

Additional file 1 **This file contains additional information regarding the validation of the microarrays and high-throughput sequencing by RT-qPCR, the phenotypes generated by larval RNAi, analyses of historical DNA methylation and alternative splicing and GC content of differentially expressed genes.** This file also contains additional materials and methods, oligonucleotide primer sequences, and detailed tables of gene ontology categories. Click here for file

Additional file 2 This file contains lists of differentially expressed genes, and associated information, based on microarray analysis. Click here for file
