# Supplementary material for: Biased gene expression in early honeybee larval development
Source: BMC Genomics. 2013 Dec 19;14:903. doi: 10.1186/1471-2164-14-903 (PMC3878232; doi:10.1186/1471-2164-14-903)
Supplement: Additional file 1 — This file contains additional information regarding the validation of the microarrays and high-throughput sequencing by RT-qPCR, the phenotypes generated by larval RNAi, analyses of historical DNA methylation and alternative splicing and GC content of differentially expressed genes. This file also contains additional materials and methods, oligonucleotide primer sequences, and detailed tables of gene ontology categories. [file 1471-2164-14-903-S1.pdf]

Additional File 1: Description of Contents.

Quality control analysis for microarray, HTS and RT-qPCR (pages 1-2).

This section contains information regarding the validation of microarray data and high-throughput sequencing data with RT-qPCR

Additional file 1: Figure S1:Validation of microarrays. (A) Validation of a selection of differentially expressed candidate genes identified by microarray analysis and tested with RT-qPCR. Genes are identified by GB numbers followed by the time-point where expression was tested in brackets. Genes for which a significant difference in expression was confirmed are marked by asterisks. One asterisk indicates $P < 0.05$, two indicate $P < 0.01$ and three indicate $P < 0.001$. (B) Correlation between microarrays and HTS data. (C) Correlation between microarrays and RT-qPCR data (D) Correlation between HTS data and RT-qPCR data. Normalised expression ratios were used to calculate Spearman's correlation coefficient (R) and associated P-value for each correlation.

RNAi phenotypes (pages 3-7).

This section contains information and examples of the phenotypes generated in by larval RNAi in honeybees.

Additional file 1: Figure S2: Examples of mandibles from individuals classified as queen-like or workers after RNAi.

Additional file 1: Figure S3: Examples of hind legs from individuals classified as queen-like or workers after RNAi. The red circles indicate areas that differ from the expected phenotype. The first example shows an individual with worker like bristles on the tarsus but no evidence for the corbicula on the tibia. The second individual shows some evidence of corbicula formation (the area without hair) but the rest of the tibia is queen-like.

Additional file 1: Figure S4: Examples of ovaries from individuals classified as queen-like or workers after RNAi.

Additional file 1: Figure S5: Examples of spermathecas from individuals classified as queen-like after RNAi.

Historical DNA methylation is associated with caste development in the honeybee (pages 8-10).

This section details bioinformatics analyses of historical DNA methylation amongst differentially expressed genes.

Additional file 1: Figure S6: Contrasting patterns of DNA methylation are observed between queen and worker larvae as measured by CpG[o/e]. The y-axis depicts the number of genes with the specific $CpG_{[o/e]}$ values given on the x-axis. In each case the data can be described as a mixture of normal distributions. These distributions are shown for queen in red and worker blue. Graphs show the $CpG_{[o/e]}$ distribution for (A) all of the genes represented on the microarray, (B) DEGs during early larval development (6–36 hr), (C) DEGs during mid-larval development (60 hr), (D) DEGs at 84 hr post-grafting, (E) DEGs during late larval development (108-132 hr), (F) all of the genes detected in the HTS analysis, (G) DEGs during

mid- larval development (60 hr) as detected by HTS. (H) The graph shows a difference plot with a negative number on the y-axis denoting over-representation of 'low' $CpG_{[o/e]}$ values in the DEGs and a positive number indicating over-representation of 'high' $CpG_{[o/e]}$ values in the DEGs at time points throughout larval development (red bars = queens, blue bars = workers). ‡ value could not be determined for DEGs in workers during late development as workers display a unimodal distribution of $CpG_{[o/e]}$ values at this time point. Statistical significance was determined using a Fisher's exact test, *** $P < 0.0001$.

Historical DNA methylation is not associated with alternative splicing during caste development in the honeybee (pages 10-11).

This section explores the relationship between GC content, rate of evolution and caste-biased expression of genes.

Additional file 1: Figure S7: The y-axis depicts the number of genes with the specific $CpG_{[o/e]}$ values given on the x-axis. In each case the data can be described as a mixture of normal distributions. These distributions are shown for genes that encode a single transcript (black lines) and genes that encode multiple transcripts (green lines). Graphs show the $CpG_{[o/e]}$ distributions for (A) all genes that are more highly expressed in queens (irrespective of whether individual variants are differentially expressed), (B) genes that are more highly expressed in queens (considering only differentially expressed variants), (C) all genes that are more highly expressed in workers (irrespective of whether individual variants are differentially expressed), D) genes that are more highly expressed in workers (considering only differentially expressed variants).

GC content and evolution (page 12)

This section explores the relationship between GC content, rate of evolution and caste-biased expression of genes.

Additional file 1: Figure S8 Genes expressed differently during queen and worker development have different GC content. Over- or under- representation of high GC content genes amongst the DEGs (relative to all of the genes found on the microarray or detected by high throughput sequencing). * $P < 0.05$, ** $P < 0.01$, *** $P < 0.001$.

Additional file 1: Supplementary methods (pages 13-14)

This section provides more detailed information regarding the methods used for RNAi, statistical analysis and determination of historical DNA methylation status for differentially expressed genes.

Additional file 1: Supplementary tables 1 – 6 (pages 15-26)

Additional file 1: Table S1: Primer sequences and efficiencies for RT-qPCR.

Additional file 1: Table S2: Number of differentially expressed genes that have orthologs in *Drosophila melanogaster.*

Additional file 1: Table S3: Families of differentially expressed genes that have a role in caste development.

Additional file 1: Table S4: Gene ontology terms that are enriched during caste development.

Additional file 1: Table S5: Pathways that are enriched during caste development.

Additional file 1: Table S6: Expression of the hexamerin genes during larval development

Additional file 1: Supplementary references (pages 27-28)

Supplementary results and figures

Quality control analysis for microarray, HTS and RT-qPCR

Microarrays are valuable tools for gene expression analysis, generating expression data for thousands of genes in a single experiment. They do however have some pitfalls. High levels of background hybridisation can reduce the range over which microarrays can accurately detect changes in gene expression [1]. Furthermore microarray probes can differ in their hybridisation properties and in some cases cross-hybridisation can occur. Finally errors can also be introduced during data analysis. This is especially true if a multiple testing correction is not used. A multiple testing correction was not performed in this analysis as performing any type of multiple testing correction eliminated all candidate genes. In this case it was essential to use other methods to validate the genes identified by the microarrays.

RT-qPCR is considered to be the gold standard for gene expression analysis [2], so it was used to confirm differential expression of a selection of candidate genes identified by the microarrays. Supplementary figure S1A. indicates the relative expression values for the candidate genes. Genes that were significantly differentially expressed between queens and workers are marked with asterisks. Of a total of 39 genes, 16 were confirmed to be significantly differentially expressed between queens and workers. A further two genes were previously published [3] bringing the total to 18 out of 41 genes (44%). This analysis indicates that less than 50% of the genes identified by the microarrays may actually have a role in caste development.

A second quality control analysis was performed using the microarray, HTS data and RT-qPCR data. A correlation analysis was performed by selecting genes that had a two-fold or greater difference in expression between the two castes. The spearman's correlation coefficient (R) was used to determine the strength of the correlation between each of the data sets. Supplementary figure S1B. shows a correlation coefficient of 0.65 between the microarray and RT-qPCR data. The HTS and RT-qPCR have the strongest correlation coefficient of 0.96 (Supplementary figure S1C.) and the HTS and microarray data have a correlation coefficient of 0.85 (Supplementary figure S1D.)

The low R value between the microarray and RT-qPCR data may be a result of the reduced dynamic range of microarrays. Microarray platforms have a tendency to underestimate fold change compared to RT-qPCR [1, 4]. Our correlation results are similar to previous findings which obtained R values of 0.71 when comparing microarray and RT-qPCR data [5] and 0.73 when comparing microarray and HTS data [1].

Although only 44% of the candidate genes confirmed with RT-qPCR the correlation between data sets was good. This indicates that while single genes may not confirm the data set as a whole is satisfactory. This justifies the use of data from the microarrays for large scale analyses such as pathway, GO analysis or CpG o/e analysis. Individual genes will require confirmation with RT-qPCR to confirm they are differentially expressed before being investigated in functional studies.

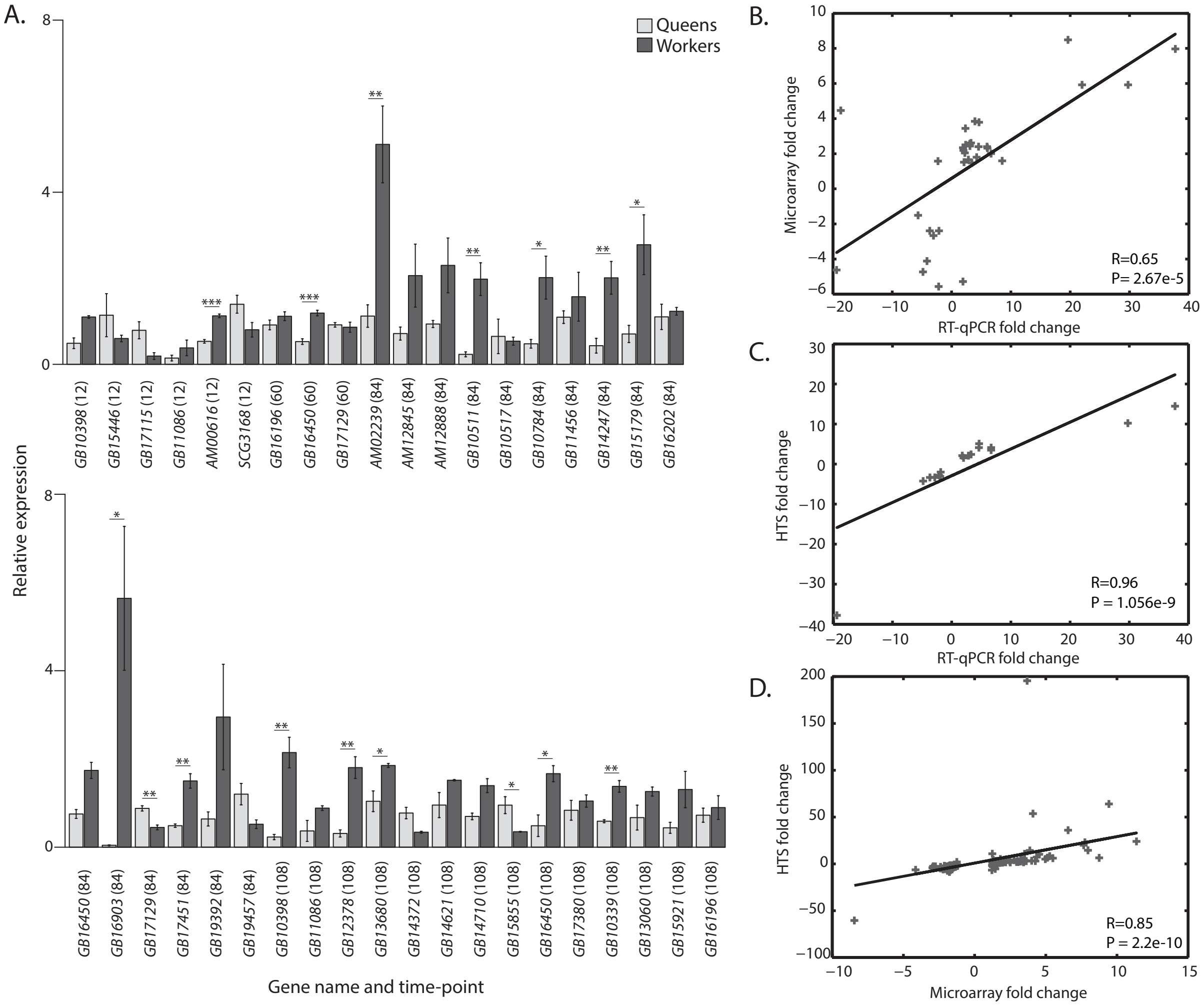

Figure S1: Validation of microarrays. (A) Validation of a selection of differentially expressed candidate genes identified by microarray analysis and tested with RT-qPCR. Genes are identified by GB numbers followed by the time-point where expression was tested in brackets. Genes that showed a significant difference in expression between queens and workers when tested with RT-qPCR are marked by asterisks. * $P < 0.05$, ** $P < 0.01$ and *** $P < 0.001$. (B) Correlation between microarray and RT-qPCR data. (C) Correlation between HTS and RT-qPCR data (D) Correlation between HTS data and microarray data. Normalised expression ratios were used to calculate Spearman's correlation coefficient (R) and associated P-value for each correlation.

RNAi phenotypes

Individuals that emerged after injection with dsRNA against *hexamerin 70b* or *egfp* were classified as either queen-like or workers based on the presence of absence of a number of characteristics. Adult queens have claw-like mandibles whereas the mandibles of adult worker bees are smooth (see Supplementary figure S2). Queen-like individuals had varying degrees of curvature in their mandibles. This variation correlates well with developmental time. Individuals that had the shortest developmental time had stronger queen-like features. The photos are arranged with the most queen-like individuals in the top row and individuals with less curvature in the last row.

The legs were also used to classify individuals as queens or workers. The tibia and tarsus on the hind legs are the most phenotypically useful as the tibia of workers contains the corbicula and the tarsus has thick bristles neither of which are present on the legs of queens. Supplementary figure S3 shows the legs of queen-like and worker individuals. Queen-like legs are covered with fine hairs and do not show evidence of the corbicula or bristles. These photos are arranged in the same way as the mandibles with individuals with the strongest queen-like phenotype at the start and the last two photos in the bottom row showing features of both queen and worker legs. These features are highlighted by red circles. The first individual has bristles on one leg but there is no evidence of corbicula formation on the other leg. The last individual shows some evidence of corbicula formation with the area circled in red lacking hair but the rest of the leg has hair on it resembling a queen-like leg.

Another key phenotypic marker of queens is their large ovaries. Supplementary figure S4 indicates examples of queen-like ovaries and worker ovaries. As above photos are arranged to show the individuals with the most queen-like phenotype first. Queen-like individuals have large ovaries with hundreds of ovarioles. Workers tend to have smaller ovaries with less than 10 ovarioles. There is some variation in worker ovary size as indicated by the last two individuals.

The final feature used to classify individuals was the presence or absence of the spermatheca. Supplementary figure S5 shows examples of spermathecas found in queen-like individuals. Workers either did not have spermathecas or they were very small.

To be classified as queen-like individuals were required to resemble queen-like individuals in three of the four characteristics.

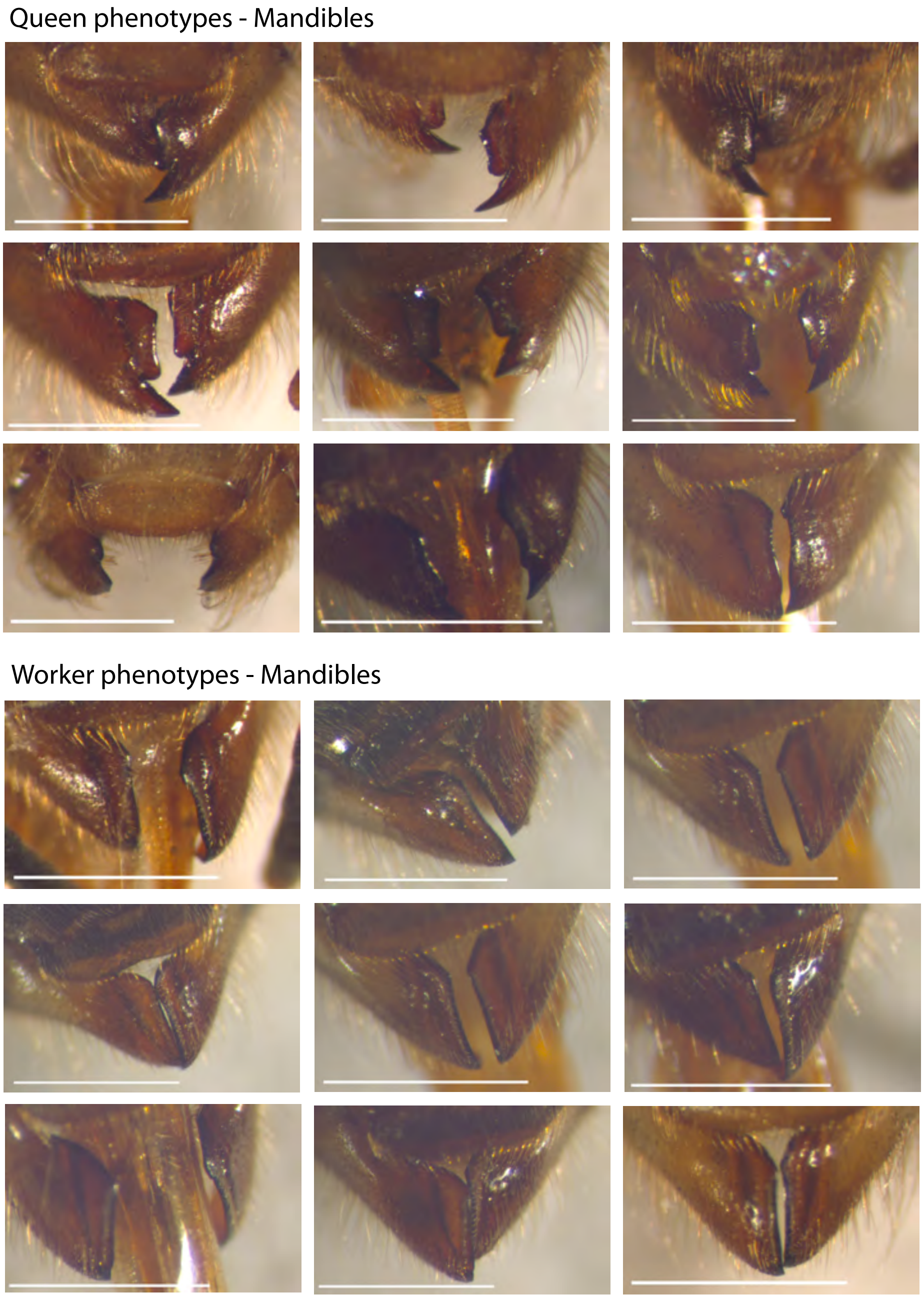

Figure S2: Examples of mandibles from individuals classified as queen-like or workers after RNAi.

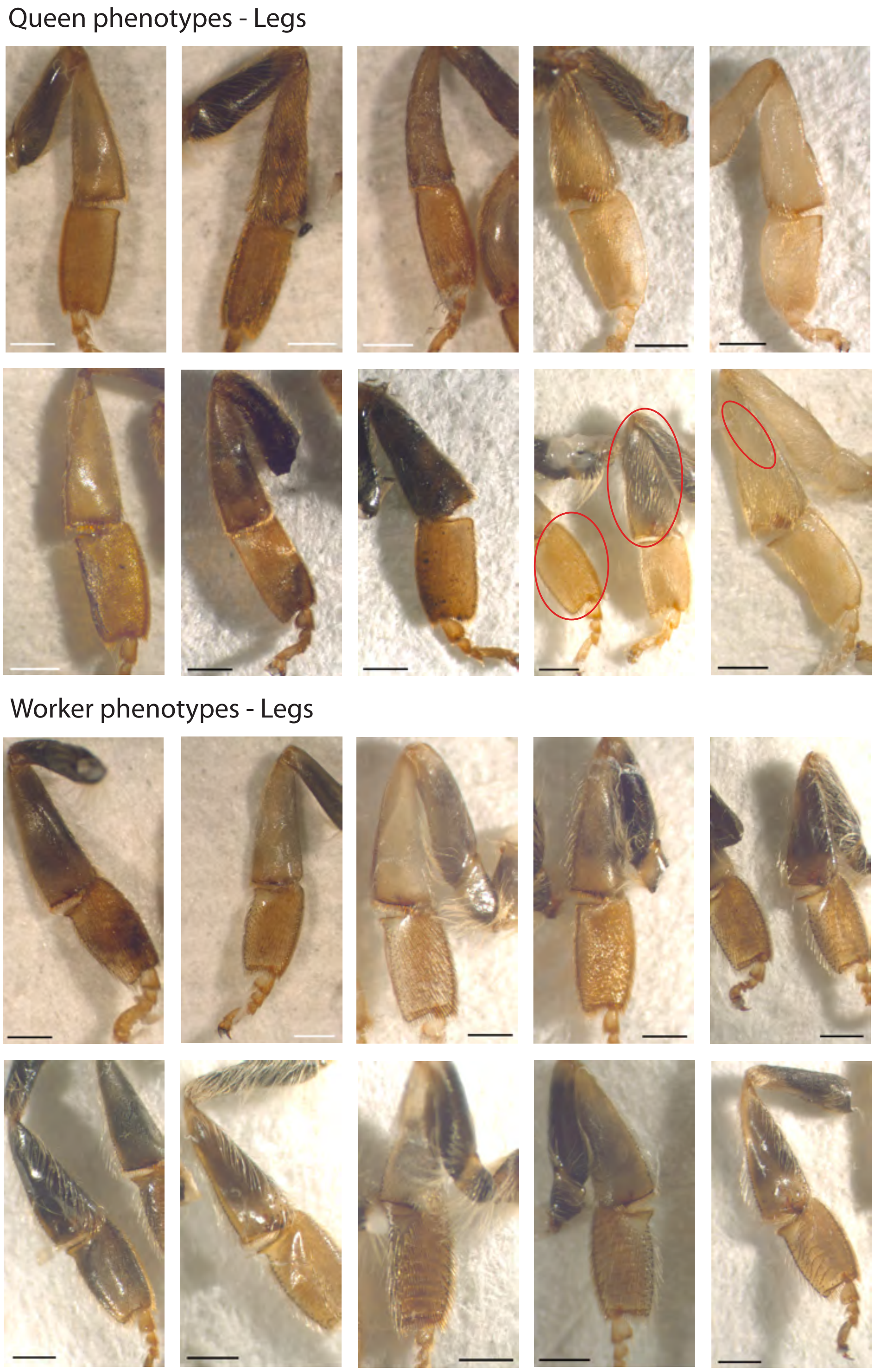

Figure S3: Examples of hind legs from individuals classified as queen-like or workers after RNAi. The red circles indicate areas that differ from the expected phenotype. The first example shows an individual with worker like bristles on the tarsus but no evidence for the corbicula on the tibia. The second individual shows some evidence of corbicula formation (the area without hair) but the rest of the tibia is queen-like.

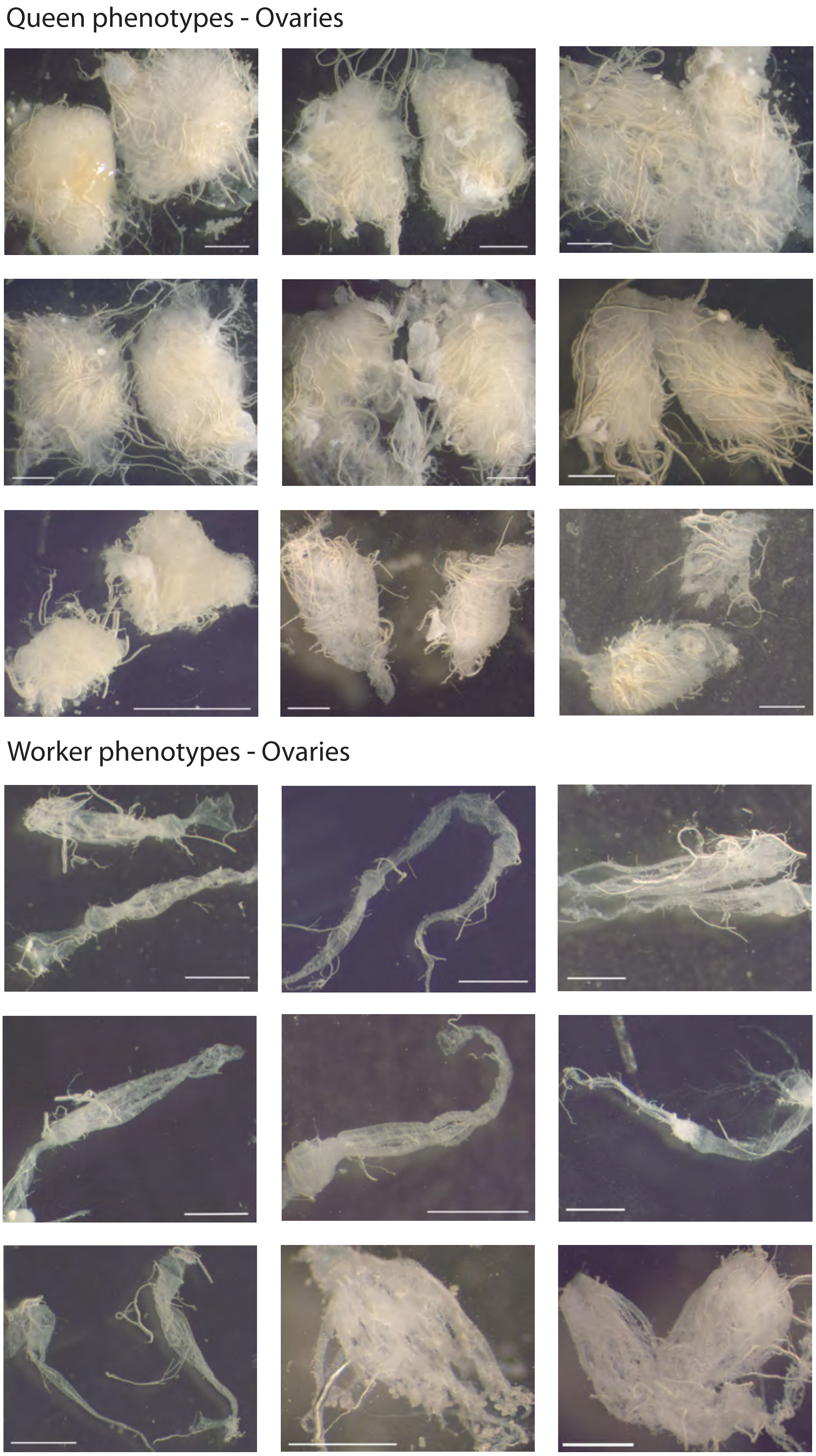

Figure S4: Examples of ovaries from individuals classified as queen-like or workers after RNAi.

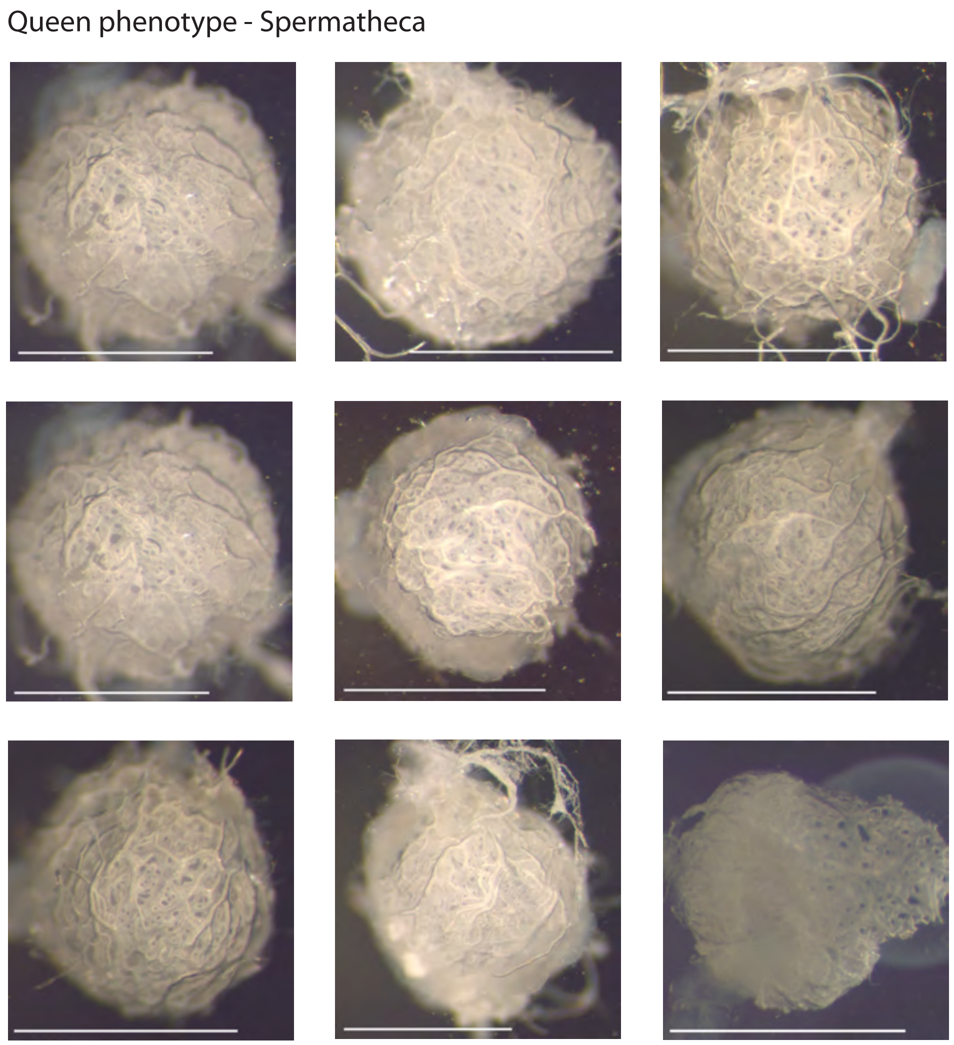

Figure S5: Examples of spermatheca from individuals classified as queen-like after RNAi.

Historical DNA methylation is associated with caste development in the honeybee.

The CpG[o/e] is a measure of the ancestral methylation state of a gene [6] as 5-methylcytosines become deaminated over evolutionary time to thymines [7], leaving a signature of low CpG content in methylated gene bodies. The CpG[o/e] ratio does not provide information on the current methylation state of a gene, although several studies have found good correlation between genes with low CpG[o/e] values and high levels of actual DNA methylation [8–10].

In the genomes of animals with DNA methylation deamination of methylated cytosine residues over evolutionary time leads to a set of genes that are depleted in the CpG content - and were historically methylated and a set of genes that are not depleted (not historically methylated). Plotting a frequency graph for the CpG[o/e] values for every gene in the genome of an animal with DNA methylation produces a bimodal distribution (actually a mixture of overlapping normal distributions), while doing the same analysis for an animal that lacks DNA methylation produces a unimodal distribution [6]. We analyzed differences in the CpG[o/e] frequency distributions of our differentially expressed genes using mclust model based clustering [11] and statistical significance between the distributions was assessed using the Fisher's exact test. A difference plot summarizing the deviations from the expected distribution for queen and worker larvae at each point during development is shown in Supplementary figure S6H.

Analysis of the CpG[o/e] ratio for all the genes detected by the microarrays produces a mixture of two normal distributions with peaks at ratios of 0.5 and 1.09 (high and low CpG[o/e] respectively, Supplementary figure S6A). During early larval development (6-36 hr), CpG[o/e] values of genes expressed in workers and queens are different, but both maintain a bimodal distribution (Supplementary figure S6B). Genes more highly expressed in queens tend to have lower CpG[o/e] values (means of 0.44 and 0.86) than those in workers (means of 0.52 and 1.13).

The trend for genes that are more highly expressed in queens to have lower CpG[o/e] values continues through to 60 hr of development (means of 0.39 and 0.95 in queens, and 0.55 and 1.13 in workers) (Supplementary figure S6C) but by 84 hours this trend switches, with genes expressed more highly in workers having lower CpG[o/e] values (0.51 and 1.09) than queens (0.56 and 1.13) implying higher levels of DNA methylation (Supplementary figure S6D.).

In late development (108-132 hours), genes more highly expressed in queens have lower CpG[o/e] ratios, but genes more highly expressed in workers fall into a unimodal distribution (means 0.42 and 0.96 for queens, 0.84 for workers, Supplementary figure S6E).

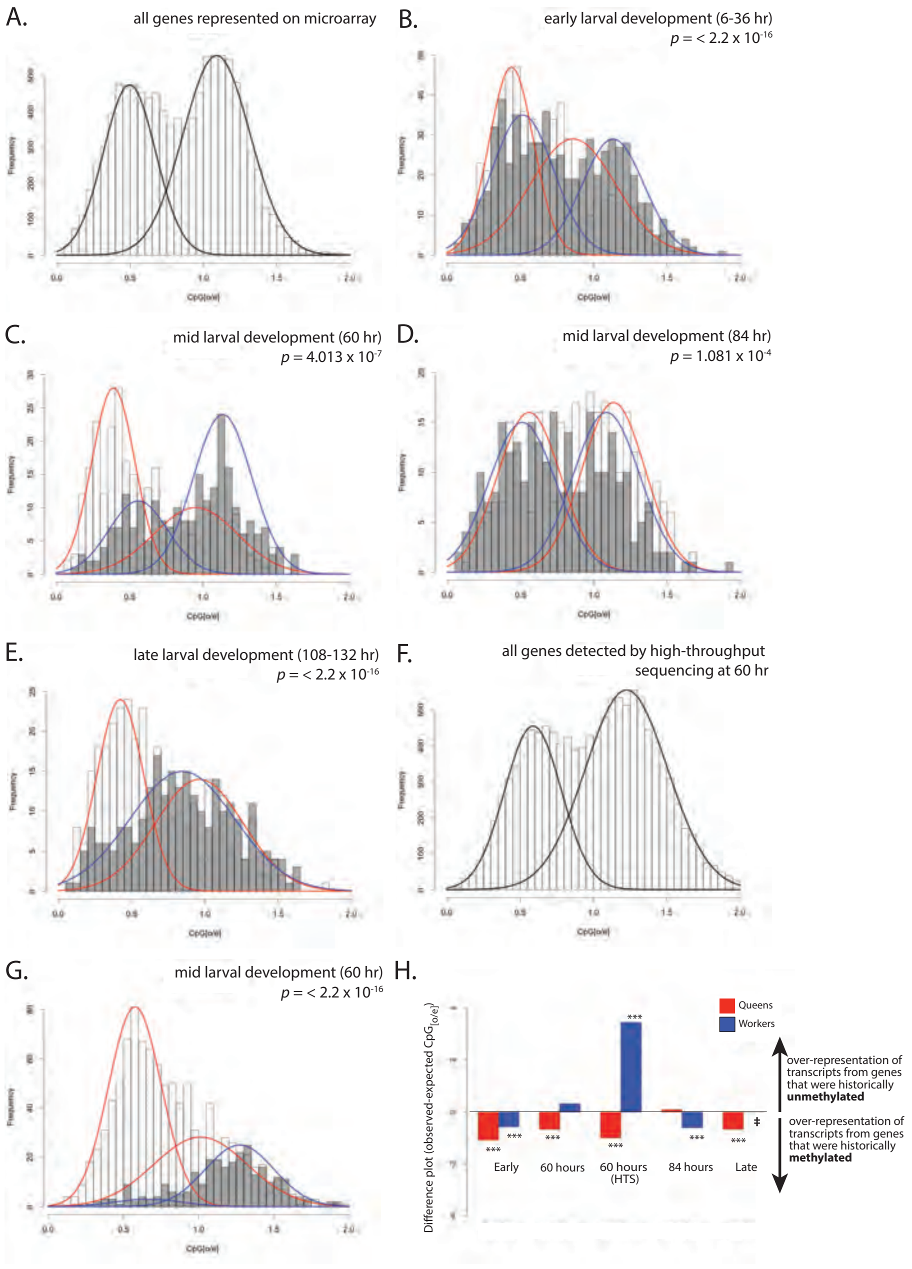

Figure S6: Queens and workers show contrasting patterns in CpG[o/e] distribution for caste specific genes. The y-axis depicts the number of genes with the specific CpG[o/e] values given on the x-axis. In each case the data can be described as a mixture of normal distributions. These distributions are shown for queen in red and worker blue. Graphs show the CpG[o/e] distribution for (A) all of the genes represented on the microarray, (B) DEGs during early larval development (6–36 hr), (C) DEGs during mid-larval development (60 hr), (D) DEGs at 84 hr post-grafting, (E) DEGs during late larval development (108-132hr), (F) all of the genes detected in the HTS analysis, (G) DEGs during mid- larval development (60 hr) as detected by HTS. (H) The graph shows a difference plot with a negative number on the y-axis denoting over-representation of low CpG[o/e] values in the DEGs and a positive number indicating over-representation of high CpG[o/e] values in the DEGs at time points throughout larval development (red bars= queens, blue bars = workers). ‡ value could not be determined for DEGs in workers during late development as workers display a unimodal distribution of CpG[o/e] values at this time point. Statistical significance was determined using a Fishers exact test, *** $P < 0.0001$.

Analysis of our high-throughput sequencing data, from 60 hr of development, yields results consistent with our array data. All the genes detected give a bimodal distribution with means at 0.59 and 1.23 (Supplementary figure S6F) and examining differences between worker and queen transcripts shows is consistent with, but more pronounced than, our microarray derived data. 50% of queen transcripts fall into the low (mean 0.58) distribution, and 50% in the high distribution (mean 1.01), while in workers, 17% fall in a low distribution, but with a higher mean that queens (0.66), and the remainder in a high distribution with a mean of 1.27 (Supplementary figure S6G).

Examining the difference between the CpG[o/e] ratios in queen and worker derived transcripts over larval development shows that queens consistently up-regulate transcripts with low CpG[o/e] ratios, implying that they activate more historically methylated genes than workers. Workers have more dynamic patterns of methylation, with a switch in mid development to transcripts with lower CpG[o/e].

Historical DNA methylation is not associated with alternative splicing during caste development in the honeybee.

To determine if patterns of alternative transcription are related to historical DNA methylation, we examined the CpG[o/e] ratios of all DEGs stratified by whether these DEGs encode a single or multiple transcripts (Supplementary figure S7A,C). In both queens and workers genes that encode multiple transcripts in larvae have higher CpG[o/e] ratios than those that encode a single transcript; queen means 0.55 and 0.98 for single transcripts and 0.62 and 1.11 for multiple transcripts, worker mean 1.17 for single transcripts and 0.63 and 1.36 for multiple transcripts. If splice variants that are not differentially regulated are excluded from this analysis we see the same trend; genes that encode a single differentially regulated splice variant have lower mean CpG[o/e] ratios (means 0.59 and 1.02) than those that express multiple differentially regulated transcripts (means 0.74 and 1.34). This analysis is only statistically significant in queens likely due to the small sample sizes in workers (Supplementary figure S7B,D).

These analyses indicate that while both queens and workers both up-regulate genes that have the capacity to encode multiple splice variants usually the transcription of only a single variant is affected. In cases where multiple transcripts are coordinately regulated these genes have a high CpG[o/e] distribution (and by inference, low levels of historical DNA methylation). This indicates that historical DNA methylation is not associated with alternative splicing during larval development in the honeybee.

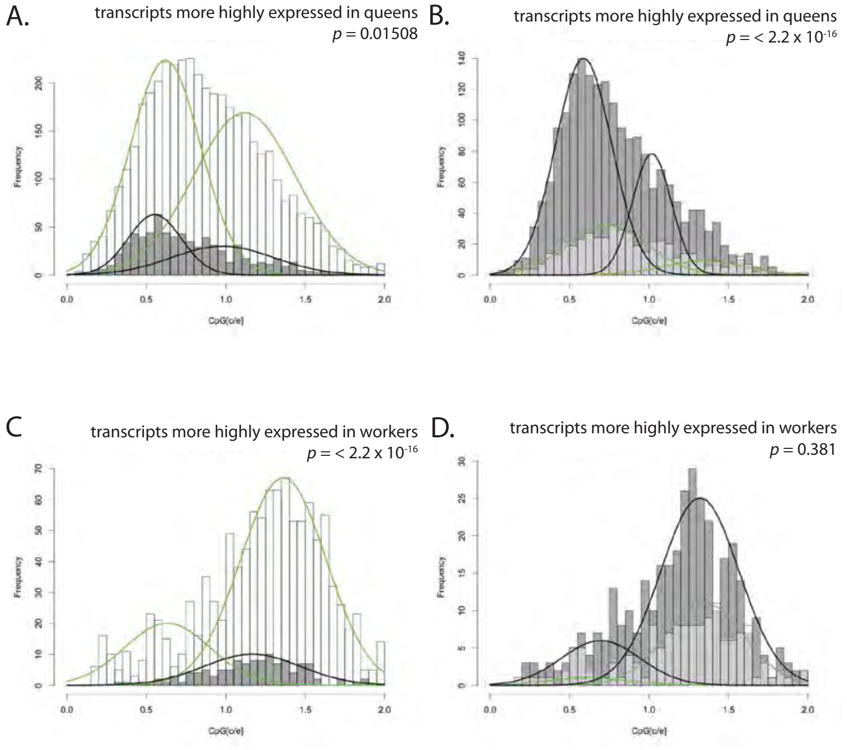

Figure S7: The y-axis depicts the number of genes with the specific CpG[o/e] values given on the x-axis. In each case the data can be described as a mixture of normal distributions. These distributions are shown for genes that encode a single transcript (black lines) and genes that encode multiple transcripts (green lines). Graphs show the CpG[o/e] distributions for (A) all genes that are more highly expressed in queens (irrespective of whether individual variants are differentially expressed), (B) genes that are more highly expressed in queens (considering only differentially expressed variants), (C) all genes that are more highly expressed in workers (irrespective of whether individual variants are differentially expressed), D) genes that are more highly expressed in workers (considering only differentially expressed variants).

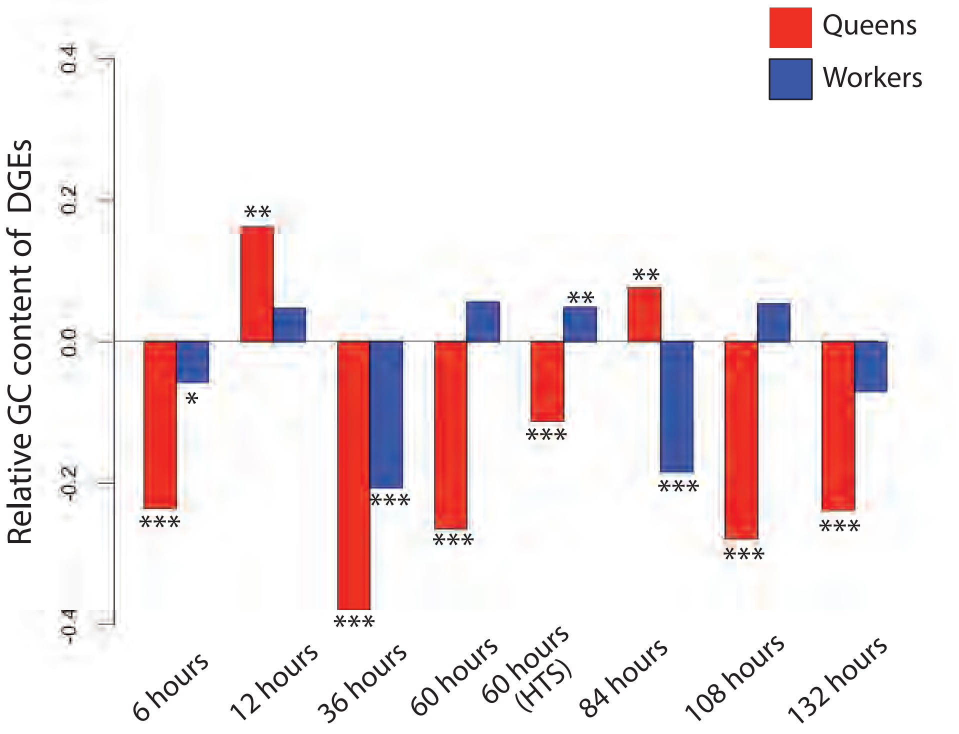

Figure S8: Genes expressed differently during queen and worker development have different GC content. Over- or under- representation of high GC content genes amongst the DEGs (relative to all of the genes found on the microarray or detected by high throughput sequencing). * $P < 0.05$, ** $P < 0.01$, *** $P < 0.001$.

GC content and evolution

Genes with high GC content can occur due to biased gene conversion owing to recombination [12] and previous studies have shown that such genes have high rates of molecular evolution and are associated with worker-biased gene expression [13, 14]. Analysis of the GC content of genes differentially expressed between castes during larval development (Supplementary figure S8) reveals that at the majority of time points both castes are biased towards differential expression of genes with a low GC content, and lower rates of sequence evolution [13]. At 12 hours and 84 hours of queen larval development, genes that are more highly expressed have a higher than expected GC content (Supplementary figure S8). In contrast genes that more highly expressed at 60 hours (as detected by HTS) have a higher than expected GC content in worker-destined larvae. This may indicate that at these specific time-points, genes that are expressed in a caste specific manner are faster-evolving, and may be important for the evolution of new traits associated with caste development.

Supplementary methods

RNAi and larval rearing

Larval injections were performed with a needle created from borosilicate capillary glass tubes (Warner Instruments). Needles were placed in a Singer MK1 Micromanipulator (Singer Instruments) and a PLI-100 Pico Injector (Harvard Apparatus) was used to deliver the injections at a pressure of 60 kPa. All larval manipulations were performed in a heated and humidified chamber. L2-L3 larvae were removed from the comb and injected with 25 ng of dsRNA. Larvae were then placed in 24 well cell culture plates (Griener), each well containing 200 μl of larval diet (18% fructose (D(-) Sigma-Aldrich), 18% glucose(D(+) Sigma-Aldrich), 3% granulated yeast (Sigma-Aldrich) solution (60%) and royal jelly (Exportim, Australia) (40%). The cell culture plates were stored in an incubator at 34 °C with a saturated solution of potassium sulphate. Larvae were fed daily with 50 -100 μl of larval diet and dead larvae were removed. When larvae stopped feeding they were transferred to 60 x 15 mm Petri dishes lined with nylon mesh. On emergence the phenotype of each individual was characterised as queen-like or worker based on the shape of the mandibles, presence of hair on the legs, number of ovarioles and the presence of a spermetheca.

RT-qPCR

1 μg of RNA from the 12 hr, 60 hr, 84 hr and 108 hr time-points was used to make cDNA using Superscript VILO^{TM} according to manufacturer's instructions. Oligonucleotide primers were designed using Primer3 [15] and Amplify [16] to span introns (where possible). The primer set and amplification efficiency for each gene is supplied in supplementary table 1. Quantitative RT-PCR, normalization and data analysis was performed as described in Cameron *et al.* 2013 [3].

Statistical analyses

Fisher's exact and χ^2 tests were performed using R, which is a statistical analysis and graphics software environment (R Development Core Team 2008). P values < 0.05 were considered to be significant.

R was also used to determine the Spearman's correlation coefficients for the quality control analysis (Supplementary figure S1B-D). Log expression ratios were obtained for genes that showed a two-fold change in expression between the castes in both the microarray and HTS data sets. Normalised expression ratios were calculated for the RT-qPCR data using the Pfaffl method as mentioned previously.

The expression ratios from each data set were graphed as linear regression plots using Matlab ([17]). As very few genes were tested with RT-qPCR at the 60 hr time-point, genes from the surrounding time-points (84 hr and 108 hr) were compared to the 60 hr HTS data in the correlation analysis. The technologies were compared in pairwise analyses in R to determine the Spearman's correlation coefficient and P value.

CpG[o/e] and GC content analysis

CpG[o/e] values for the microarray data were calculated using the sequence data file (Official GeneSet_Array.txt) [18]. The CpG[o/e] values for the HTS data were calculated using sequence data from the coding regions from *A. mellifera* genes (Amel_4.5, NCBI). Sequence statistics were generated using the Geomatrix software suite [19]. CpG[o/e] values were calculated from these statistics using the calculation described by Elango *et al.* (2009) [6]. The distributions of CpG[o/e] within honeybee genes can be described as a mixture of overlapping normal distributions [6]. The number of components in these distributions was estimated in R (www.r-project.org) using mclust [11] model-based clustering. The best fitting model was identified among several non-nested models using Bayesian information criteria (BIC). The Fishers exact test was used to determine whether there were differences between samples in the numbers of genes classified under each of the distributions identified in mclust. GC content was calculated from the nucleotide composition of honeybee coding genes. Genes with a GC content of $> 38\%$ were classified as GC rich and genes with a GC content of $< 38\%$ were classified as GC poor [13]. The occurrence of GC rich and GC poor genes within our differentially expressed gene lists was compared with the expected occurrence based on GC content of all the genes on the microarray or detected as expressed by high throughput sequencing using a χ^2 test.

Supplementary tables

Table S1: Primer sequences and efficiencies for RT-qPCR

Gene identifier	Forward primer	Reverse primer	Efficiency	Amplicon size
GB10398	TGATTGAATATCAACGAGAGAGTCC	TCGTGCTTGTAATGATTTTGATTG	2.086	149
GB15446	TTCGCGAACTCCTCGTACCT	AACTTCCGCTGGCAAATCTC	2.091	86
GB11715	GCAAATGCTCATGGTGTTCC	TTTCCCTTTGAGCTGCGATT	2.13	130
GB11086	GAAATTGAAAGAGCAACTGATGAAA	TCGTTGTCGCAATAAACCTTC	1.975	138
BH10040L09	ACCTCATTTGGTTATGTTCTTCAAA	CACGTTGCCATTCAAAATTTTATTAC	1.915	118
GB11252	CGTGTTACCGGCAGCTCAAT	ATCAGCCAGGCATCCCCATA	2.004	118
GB16189	TCGAACCAAGATGGTACTGGAA	TTGTTGTGCTTGCAGTCGTG	1.928	100
GB16450	TGGTTTCGAAGTGAAGATCTATAAGG	CGCAGATACATTATCACCGATTTT	1.988	148
GB17129	GTGTGAAAACAAAGTGCGTGTC	TGCATTTGTTGAAGTTTTGAGG	2.094	145
BB160017A10G05	GGTTGGTGTAACATTAAATTTGTTCG	CGGTCAACGTGACACATTACAA	2.087	139
BQ40010J21	TGGAACAAATATTTCGCTCACTG	CGCCACAAGCTAAATACGTTCG	2.055	139
BQ30029D08	TGGAACAAATATTTCGCTCACTG	CGCCACAAGCTAAATACGTTCG	1.956	139
GB10511	CGGTCACAAGGAGTTGCTGT	GTCCCAGTCGAGCGTTTTTA	1.936	154
GB10517	GCATTGCAGCGAAAGAGAGA	ATTTCCCGCGTTATTTCGTC	1.904	141
GB10784	GGCTCGGATGATATGACCAC	GGCAAGTTCCGTTACCCTTT	1.95	138
GB11456	CAGGCAGCTTTAATGGCAAG	TCCTGATCCATTTCCTAAAAGTCC	2.007	144
GB14247	TCTCGACGATGAGAATTTACAATATGA	GGTTTGGCGTGTTCCAAAAG	1.976	150
GB15179	GAGGCAGTGCCCTTATCCTG	TGGCTTCCCCTCTGTGATTT	2.001	97
GB16202	CGCTTCGGATAAAATGCTGA	ACCGCGTCAAATCCATCTCT	1.949	150
GB16903	AGCGATAGCCAATGCTGATG	GGCTGAGGCAAATCTCCTTG	2.064	126
GB17451	CATGATGGTGGTTGTGTTCCT	GTGGAGCTTCTCCATAAATCCAT	1.902	100
GB19392	TCTATTTTATTGTCGGCCTTATCTTC	CGGCACGTTCTTCATTCTCA	1.915	108
GB19457	ACTCGAGGAGACGAATGTGC	CGATGTTGTTGTTGTCGTAGGG	1.988	118
GB10398	TGATTGAATATCAACGAGAGAGTCC	TCGTGCTTGTAATGATTTTGATTG	2.086	149
GB12378	TCATCGTTACGCTCGGTCTTT	TCCAATGCGGTGGAAACATA	1.993	142
GB13680	GTTGCAGCGTTTGAAGGACA	CAGGTGCAATTGTAACAACACG	1.922	130
GB14372	GGCGTTGAATTCGAGGATGT	GCCAGGTAACGGCAAATAGG	2.053	146
GB14621	GCACCTAGGAAGGAGCAACC	CCGTCTGCTCGATGGTGATA	1.997	61
GB14710	GCCTGGCGCAGAAAAATATAA	AATTCATTTCCCACGAACCA	1.965	60
GB15855	TTTACCAAATTAAGAATGCCAGTGA	TGCATGGACCACACCACATA	1.912	102
GB17380	GGTCATGTGCTGGCGAACTA	CCGAATTGGGACACCTCTTC	2.11	198
GB10339	GCACGATTCAACTCGACAATAA	CCGACCGTCTTCACCGTAAT	1.9118	91
GB15921	GTTCGACTCTTGGCCTTGGT	TTTCGGAACTCTGGAAGCAAA	2.06	152
GB16196	GCAATTCAAAACTTCTAATGATGTCG	GATGTCCTGGGAGAAGTGAGAA	1.935	135

Table S2: Number of differentially expressed genes that have orthologs in *Drosophila melanogaster*

	**Microarray**								**HTS**
	6 hr	12 hr	36 hr	60 hr	84 hr	108 hr	132 hr	Total	60 hr
**Queens**									
With orthologs	400	63	143	285	296	232	105	1524	1825
Without orthologs	42	13	11	42	34	18	10	170	486
**Workers**									
With orthologs	436	77	143	233	268	149	111	1417	303
Without orthologs	120	22	13	71	94	48	49	417	154

Table S3: Families of differentially expressed genes that have a role in caste development

Genes involved in the response to reactive oxygen species

Gene identifier	Gene symbol and name	Time-point	Fold change	Caste
GB19380	*Tpx-1 thioredoxin peroxidase 1*	6 hr, 60 hr, 84 hr, 108 hr, 60 hr HTS	1.44, 1.6, 2.01, 1.63, 2.3	Q, Q, Q, Q, Q
GB15855	*Trx-2 thioredoxin 2*	60 hr, 108 hr, 60 hr HTS	1.56, 2.39, 2.8	Q, Q, Q
GB12870	*Trx/Gtx thioredoxin/glutaredoxin protein*	6 hr, 60 hr, 108 hr, 60 hr HTS	1.36, 1.8, 1.37, 2.1	Q, Q, Q, Q, Q
GB30227	*Cat catalase*	84 hr, 108 hr	4.73, 1.92	Q, Q
GB18045	*GstD1 glutathione S-transferase D1*	6 hr, 12 hr, 84 hr, 108 hr	3.1, 1.42, 1.42, 1.61	Q, W, Q, Q
GB14372	*GstS4 glutathione S-transferase S4*	6 hr, 84 hr, 132 hr	1.34, 1.38, 3.72	W, W, Q
GB15512	*GstU1 glutathione S-transferase U1*	6 hr, 60 hr, 84 hr, 108 hr, 60 hr HTS	1.71, 1.79, 3.01, 2.64, 2.7	W, W, W, W, W

Genes involved in programmed cell death

Gene identifier	Gene symbol and name	Time-point	Fold change	Caste
GB13004	*LOC408851 putative cysteine proteinase CG12163-like*	60 hr, 108 hr	2.12, 2.3	W, W
GB19923	*LOC552756 cathepsin L-like*	84 hr, 108 hr, 132 hr	1.47, 1.43, 1.63	W, W, Q
GB18912	*LOC409517 counting factor associated protein D-like*	60 hr, 84 hr, 108 hr, 132 hr, 60 hr HTS	4.00, 2.49, 2.34, 1.58, 3.4	W, W, W, Q, W
GB16903	*cathD cathepsin D*	60 hr, 84 hr, 108 hr, 60 hr HTS	4.54, 5.93, 2.66, 10.2	W, W, W, W
GB19916	*SCR-B9 scavenger receptor class B, type 9*	84 hr, 108 hr	2.42, 2.77	W, W
GB16388	*SCR-B8 scavenger receptor class B, type 8*	6 hr, 36 hr	1.45, 1.56	W, W
GB12378	*LOC100577943 lysosome membrane protein 2-like*	60 hr, 108 hr	2.15, 2.32	W, W
GB17541	*LOC409667 BCL2/adenovirus E1B 19 kDa protein-interacting protein 3-like*	6 hr, 60 hr, 84 hr, 108 hr, 132 hr, 60 hr HTS	1.61, 1.63, 2.56, 1.89, 1.23, 1.36, 1.7	W, W, W, W, W, W, W
GB16450	*LOC411381 caspase-like*	12 hr, 60 hr, 84 hr	1.26, 2.05, 1.48	W, W, W
GB10784	*LOC412885 protein N-terminal asparagine amidohydrolase-like*	6 hr, 60 hr, 84 hr, 108 hr, 60hr HTS	1.48, 2.03, 1.80, 2.51, 2.2	W, W, W, W, W
GB17294	*LOC725570 tumor necrosis factor, alpha-induced protein 8-like protein*	6 hr, 12 hr, 84 hr, 60 hr HTS	1.24, 1.41, 1.40, 2.1	W, Q, Q, Q
GB17129	*LOC726172 e3 ubiquitin-protein ligase IAP-3-like*	84 hr	2.14	Q

Genes involved in JH synthesis, degradation or response

Gene identifier	Gene symbol and name	Time-point	Fold change	Caste
**JH synthesis/degradation**				
GB10517	*jhamt juvenile hormone acid methyltransferase*	6 hr, 84 hr, 108 hr,	1.55, 3.40, 1.80	Q, Q, Q
CYP15A1	*CYP15A1 cytochrome P450 15A1*	6 hr, 60 hr, 84 hr, 108 hr	1.21, 2.8, 6.7, 1.94	Q, Q, Q, Q
GB15327	*Jhe juvenile hormone esterase*	60 hr, 84 hr, 108 hr	3.69, 4.78, 2.18	W, W, W
**JH response**				
GB12562	*LOC408421 uncharacterised LOC408421*	6 hr, 12 hr, 60 hr HTS	1,67, 1.66, 169	W, W, W
GB19754	*LOC726672 uncharacterised LOC726672*	36 hr, 84 hr, 108 hr, 132 hr	1.56, 1.92, 2.38, 1.39	W, Q, Q, Q
GB15308	*LOC552039 uncharacterised LOC552039*	60 hr	1.87	W
GB14761	*LOC100577486 uncharacterised LOC100577486*	84 hr	2.12	Q
GB18082	*LOC724781 uncharacterised LOC724781*	108 hr	1.46	Q
GB12029	*LOC552722 glyoxalase domain-containing protein 4-like*	132 hr	1.34	Q
GB14097	*JhI-1 ribonuclease Z, mitochondrial*	60 hr HTS	1.69	Q

* Caste is indicated with Q for queen and W for worker

Table S4: Gene ontology terms that are enriched during caste development

Microarrays

**Queens**			**Workers**		
**Cluster name**	**Enrichment score**	**Time-point**	**Cluster name**	**Enrichment score**	**Time-point**
Nucleotide binding	6.3958	6 hr	Organophosphate and lipid metabolic processes	2.08	6 hr
Protein folding	5.2381	6 hr	Muscle protein	1.99	6 hr
Macromolecular complex assembly	4.9306	6 hr	Sphingolipid metabolic process	1.78	6 hr
Aminoacyl-tRNA biosynthesis	3.2726	6 hr	Male sterility, NAD-binding	1.63	6 hr
WD40 repeat	3.2611	6 hr	Component of membrane	1.57	6 hr
ATPase activity	3.1422	6 hr	Amino acid transport	1.48	6 hr
Heat shock protein	2.9571	6 hr	Isoprenoid and retinoid binding	1.17	6 hr
Organelle lumen	2.4339	6 hr	Programmed cell death	1.1	6 hr
DNA replication	2.1724	6 hr	Oxidative phosphorylation	2.92	12 hr
Aminoacyl-tRNA synthetase	1.9583	6 hr	Metabolism	1.7	12 hr
Intracellular transport	1.8214	6 hr	Cytochrome P450 activity	1.3	12 hr
Glutathione S-transferase	1.6133	6 hr	Nucleotide binding	4.46	36 hr
Regulation of translation	1.5886	6 hr	Ligase activity	2.88	36 hr
Tetratricopeptide repeat	1.5116	6 hr	Helicase activity	2.09	36 hr
Thioredoxin activity	1.4816	6 hr	xidative phosphorylation	4.88	60 hr
Nucleosome organisation and assembly	1.464	6 hr	Cytochrome p450 activity	2.34	60 hr
Helicase activity	1.4534	6 hr	Drug metabolism	1.96	60 hr
Oxidative phosphorylation	1.913	12 hr	Exopeptidase activity	1.88	60 hr
Nuclease activity	2.2786	36 hr	Serine hydrolase activity	1.69	60 hr
RNA processing and splicing	2.1778	36 hr	Component of endoplasmic reticulum	3.3	84 hr
Translation	1.5838	36 hr	Drug metabolism	1.94	84 hr
Component of mitochondria or ribosome	4.0506	60 hr	Glutathione S-transferase activity	1.77	84 hr
Components of organelle membrane or envelope	3.3021	60 hr	Cofactor binding	1.5	84 hr
Mitochondria	2.6413	60 hr	Thioredoxin activity	1.39	84 hr
RNA binding	2.0885	60 hr	Metabolic process	1.37	84 hr

Components of the cuticle	2.0103	60 hr	Peptidase activity	1.32	84 hr
Nuclease activity	1.8113	60 hr	Microsome	1.17	84 hr
Cellular respiration	2.4143	84 hr	Proteolysis	2.08	108 hr
Mitochondria	2.4023	84 hr	Hormone binding	2.06	108 hr
Protein transport	1.9434	84 hr	Ribosome and translation	1.99	108 hr
Energy generation	1.8485	84 hr	Calcium ion binding	1.3	108 hr
Heat shock proteins	1.6997	84 hr	Component of the ribosome	3.7	132 hr
Nucleosome organisation	1.4496	84 hr	Nucleosome organisation and assembly	1.29	132 hr
Macromolecular complex assembly	1.4106	84 hr			
Oxidative phosphorylation	12.4814	108 hr			
Mitochondria	4.9331	108 hr			
Energy generation	3.5888	108 hr			
Constituents of mitochondria and ribosome	3.0013	108 hr			
Membrane transport	2.2256	108 hr			
Cytochrome C oxidase activity	2.2235	108 hr			
Cofactor metabolic process	2.079	108 hr			
Endoplasmic reticulum	2.0705	108 hr			
Flavoprotein	1.8711	108 hr			
TCA cycle	1.8612	108 hr			
Metal cluster binding	1.6529	108 hr			
Signal recognition particle	1.5462	108 hr			
Response to stress	1.4696	108 hr			
Heme binding	1.4404	108 hr			
Protein targeting to the endoplasmic reticulum	1.2495	108 hr			
Proteosome	2.459	132 hr			
Proteolysis	2.1899	132 hr			
Translation	2.1281	132 hr			
Translation initiation	1.73	132 hr			
Protein folding	1.6149	132 hr			

HTS

Queens			Workers		
**Cluster name**	**Enrichment score**	**Time-point**	**Cluster name**	**Enrichment score**	**Time-point**
Translation	17.6	60 hr	Hormone binding	2.43	60 hr
Organelle lumen	14.2	60 hr	Propanoate metabolism	2.43	60 hr
Energy generation	11	60 hr	Peptidase activity	2.41	60 hr
Cell cycle	10.9	60 hr	Signal peptide	2.36	60 hr
Ribosome	10.88	60 hr	Microsome	2.28	60 hr
Mitochondria	9.15	60 hr	Extracellular matrix	1.96	60 hr
Nucleotide binding	7.92	60 hr	Muscle protein	1.92	60 hr
RNA processing	7.44	60 hr	Arginine metabolic process	1.61	60 hr
RNA processing and Splicing	7.2	60 hr	Cofactor binding	1.6	60 hr
Cellular macromolecule catabolic process	6.35	60 hr	Actin cytoskeletion	1.54	60 hr
Translation	5.44	60 hr	Drug metabolism	1.18	60 hr
Helicase activity	5.01	60 hr	Cellular amino acid catabolic process	1.16	60 hr
Ligase activity	4.96	60 hr	Calcium ion binding	1.12	60 hr
mRNA binding	4.03	60 hr	Sugar transporter	1.12	60 hr
Protein transport	3.86	60 hr	Carboxypeptidase activity	1.1	60 hr
Regulation of splicing	3.86	60 hr			
ATPase	3.72	60 hr			
Macromolecular complex assembly	3.67	60 hr			
DNA replication	3.57	60 hr			
Protein transport	3.27	60 hr			
Proteasome component region	3.25	60 hr			
Organelle outer membrane	3.18	60 hr			
mRNA transport	3.17	60 hr			
Phosphate metabolic process	2.9	60 hr			
Protein transport	2.8	60 hr			
Ubiquitin	2.73	60 hr			
Chromosome organization	2.61	60 hr			

Protein folding	2.5	60 hr
Proteasome	2.49	60 hr
Ubiquitin mediated proteolysis	2.44	60 hr
Like-Sm ribonucleoprotein, core	2.38	60 hr
DNA helicase activity	2.27	60 hr
Nucleosome organisation	2.14	60 hr
Thiolester hydrolase activity	2.11	60 hr

Table S5: Pathways that are enriched during caste development

Microarrays

**Workers**				**Queens**			
**Pathway name**	**Time-point**	**No. of genes**	**P value**	**Pathway name**	**Time-point**	**No. of genes**	**P value**
Fatty acid metabolism	12 hr	6	0.0001	Aminoacyl-tRNA biosynthesis	6 hr	13	0
Limonene and pinene degradation	12 hr	6	0.0001	Selenoamino acid metabolism	6 hr	5	0.0081
Tryptophan metabolism	12 hr	5	0.0002	DNA replication	6 hr	7	0.0149
Lysine degradation	12 hr	5	0.0006	Spliceosome	6 hr	13	0.0384
beta-Alanine metabolism	12 hr	4	0.0021	Other glycan degradation	6 hr	4	0.0394
Propanoate metabolism	12 hr	4	0.0041	Spliceosome	36 hr	7	0.0466
Butanoate metabolism	12 hr	4	0.007	Citrate cycle (TCA cycle)	84 hr	6	0.0204
Glycolysis / Gluconeogenesis	12 hr	4	0.0088	Oxidative phosphorylation	108 hr	28	0
Valine, leucine and isoleucine degradation	12 hr	4	0.0131	Citrate cycle (TCA cycle)	108 hr	7	0.0031
Metabolism of xenobiotics by cytochrome P450	12 hr	3	0.0324	Glycine, serine and threonine metabolism	108 hr	5	0.0272
Drug metabolism	12 hr	3	0.0399	Pyruvate metabolism	108 hr	5	0.0484
Glycerolipid metabolism	12 hr	3	0.048	Proteasome	132 hr	6	0.0007
Ascorbate and aldarate metabolism	60 hr	6	0	Lysosome	132 hr	5	0.0461
Limonene and pinene degradation	60 hr	7	0.0004				
Retinol metabolism	60 hr	5	0.0007				
Metabolism of xenobiotics by cytochrome p450	60 hr	5	0.0029				
Drug metabolism	60 hr	5	0.0045				
Pentose and glucuronate interconversions	60 hr	4	0.0086				
Starch and sucrose metabolism	60 hr	5	0.0107				
Glycerolipid metabolism	60 hr	4	0.041				
Androgen and estrogen metabolism	60 hr	3	0.0419				
Metabolism of xenobiotics by cytochrome p450	84 hr	5	0.0036				
Arginine and proline metabolism	84 hr	6	0.004				
Drug metabolism	84 hr	5	0.0055				
Ribosome	108 hr	9	0.0026				

Ribosome	132 hr	10	0				
**HTS**							
**Workers**				**Queens**			
**Pathway name**	**Time-point**	**No. of genes**	**P value**	**Pathway name**	**Time-point**	**No. of genes**	**P value**
Glycolysis / Gluconeogenesis	60 hr	7	0.0005	Ribosome	60 hr	47	0
Limonene and pinene degradation	60 hr	7	0.0007	Oxidative phosphorylation	60 hr	42	0
Histidine metabolism	60 hr	4	0.0009	Spliceosome	60 hr	52	0
Arginine and proline metabolism	60 hr	6	0.005	Aminoacyl-tRNA biosynthesis	60 hr	23	0.0008
Propanoate metabolism	60 hr	5	0.0067	RNA degradation	60 hr	24	0.002
Ascorbate and aldarate metabolism	60 hr	4	0.009	DNA replication	60 hr	19	0.0078
Fructose and mannose metabolism	60 hr	4	0.016	Ubiquitin mediated proteolysis	60 hr	37	0.0132
Phenylalanine metabolism	60 hr	4	0.0161	Cysteine and methionine metabolism	60 hr	12	0.0178
Starch and sucrose metabolism	60 hr	5	0.0172	Proteasome	60 hr	18	0.0203
beta-Alanine metabolism	60 hr	4	0.0301	Pyrimidine metabolism	60 hr	31	0.0382
Valine, leucine and isoleucine degradation	60 hr	5	0.0345	Nucleotide excision repair	60 hr	17	0.0466
Pentose phosphate pathway	60 hr	4	0.0358				
Metabolism of xenobiotics by cytochrome p450	60 hr	4	0.0488				

Table S6: Expression of the hexamerin genes during larval development

**Gene identifier**	**60 hr**	**60 hr HTS**	**84 hr**	**108 hr**	**132 hr**
hex70a.1	8.49 W	26.95 W	-	2.67 W	-
hex70b.1	5.11 W	6.56 W	-	-	-
hex70b.2	-	7.56 W	-	-	-
hex70c.1	7.97 W	14.46 W	-	-	4.46 W
hex110.1	-	22.59 W	-	-	4.09 W
hex110.2	-	20.07 W	-	-	-
hex110.3	-	10.23 W	-	-	-

* alternative transcripts are indicated with numerals. Gene expression information for these alternative transcripts was obtained from HTS data only

Bibliography

[1] Marioni, J.C., Mason, C.E., Mane, S.M., Stephens, M., Gilad, Y.: RNA-seq: An assessment of technical reproducibility and comparison with gene expression arrays. Genome Res. **18**(9), 1509–1517 (2008). doi:10.1101/gr.079558.108. http://genome.cshlp.org/content/18/9/1509.full.pdf+html

[2] Bustin, S.A., Benes, V., Garson, J.A., Hellemans, J., Huggett, J., Kubista, M., Mueller, R., Nolan, T., Pfaffl, M.W., Shipley, G.L., Vandesompele, J., Wittwer, C.T.: The MIQE guidelines: minimum information for publication of quantitative real-time pcr experiments. Clinical Chemistry **55**(4), 611–622 (2009). doi:10.1373/clinchem.2008.112797. http://www.clinchem.org/content/55/4/611.full.pdf+html

[3] Cameron, R., Duncan, E., Dearden, P.: Stable reference genes for the measurement of transcript abundance during larval caste development in the honeybee. Apidologie, 1–10 (2013). doi:10.1007/s13592-012-0187-0

[4] Yuen, T., Wurmbach, E., Pfeffer, R.L., Ebersole, B.J., Sealfon, S.C.: Accuracy and calibration of commercial oligonucleotide and custom cDNA microarrays. Nucleic Acids Res. **30**(10), 48 (2002). doi:10.1093/nar/30.10.e48. http://nar.oxfordjournals.org/content/30/10/e48.full.pdf+html

[5] Morey, J., Ryan, J., Van Dolah, F.: Microarray validation: factors influencing correlation between oligonucleotide microarrays and real-time PCR. Biol. Proced. Online. **8**, 175–193 (2006)

[6] Elango, N., Hunt, B.G., Goodisman, A.D. Michaels, Yi, S.V.: DNA methylation is widespread and associated with differential gene expression in castes of the honeybee, *Apis mellifera*. Proc. Natl. Acad. Sci. U. S. A. **106**(27), 11206–11211 (2009). doi:10.1073/pnas.0900301106. http://www.pnas.org/content/106/27/11206.full.pdf+html

[7] Coulondre, C., Miller, J.H., Farabaugh, P.J., Gilbert, W.: Molecular basis of base substitution hotspots in *Escherichia coli*. Nature **274**, 775–780 (1978)

[8] Lyko, F., Foret, S., Kucharski, R., Wolf, S., Falckenhayn, C., Maleszka, R.: The honey bee epigenomes: differential methylation of brain DNA in queens and workers. PLoS Biol. **8**, 1000506 (2010). doi:10.1371/journal.pbio.1000506

[9] Foret, S., Kucharski, R., Pittelkow, Y., Lockett, G.A., Maleszka, R.: Epigenetic regulation of the honey bee transcriptome: unravelling the nature of methylated genes. BMC Genomics **10**, 472 (2009)

[10] Sarda, S., Zeng, J., Hunt, B.G., Yi, S.V.: The evolution of invertebrate gene body methylation. Mol. Biol. Evol., (2012). doi:10.1093/molbev/mss062. http://mbe.oxfordjournals.org/content/early/2012/03/21/molbev.mss062.full.pdf+html

[11] Fraley, C., Raftery, A.E.: Enhanced model-based clustering, density estimation, and discriminant analysis software: Mclust. Journal of Classification **20**, 263–286 (2003). doi:10.1007/s00357-003-0015-3

[12] Duret, L., Galtier, N.: Biased gene conversion and the evolution of mammalian genomic landscapes. Annu Rev Genomics Hum Genet **10**, 285–311 (2009)

[13] Kent, C.F., Minaei, S., Harpur, B.A., Zayed, A.: Recombination is associated with the evolution of genome structure and worker behavior in honey bees. Proc. Natl. Acad. Sci. USA **109**, 18012–18017 (2012)

[14] Kent, C.F., Minaei, S., Harpur, B.A., Zayed, A.: Reply to hunt et al.: Worker-biased genes have high guaninecytosine content and rates of nucleotide diversity in the honey bee. Proc. Natl. Acad. Sci. USA **110**, 447 (2013)

[15] Rozen, S., Skaletsky, H.: Primer3 on the WWW for General Users and for Biologist Programmers. Bioinformatics Methods and Protocols. Methods in Molecular Biology, vol. 132, pp. 365–386 (1999). http://www.springerprotocols.com/Abstract/doi/10.1385/1-59259-192-2:365

[16] Engels, B.: Amplify3. http://engels.genetics.wisc.edu/amplify/

[17] MATLAB: Version 7.10.0 (R2010a). The MathWorks Inc, Natick, Massachusetts, (2010)

[18] Honeybee Oligonucleotide Microarray. http://www.biotec.uiuc.edu/centers/Keck/Functional_genomics/Honey%20Bee%20Oligo.htm

[19] Geomatrix Software Suite. http://www.genomatix.de/cgi-bin/tools/tools.pl
